# IFCN-endorsed practical guidelines for clinical magnetoencephalography (MEG)

**DOI:** 10.1016/j.clinph.2018.03.042

**Published:** 2018-08

**Authors:** Riitta Hari, Sylvain Baillet, Gareth Barnes, Richard Burgess, Nina Forss, Joachim Gross, Matti Hämäläinen, Ole Jensen, Ryusuke Kakigi, François Mauguière, Nobukatzu Nakasato, Aina Puce, Gian-Luca Romani, Alfons Schnitzler, Samu Taulu

**Affiliations:** a Department of Art, Aalto University, Helsinki, Finland; b McConnell Brain Imaging Centre, Montreal Neurological Institute, McGill University, Montreal, QC, Canada; c Wellcome Centre for Human Neuroimaging, University College of London, London, UK; d Epilepsy Center, Neurological Institute, Cleveland Clinic, Cleveland, OH, USA; e Clinical Neuroscience, Neurology, University of Helsinki and Helsinki University Hospital, Helsinki, Finland; f Centre for Cognitive Neuroimaging, University of Glasgow, Glasgow, UK; g Institute for Biomagnetism and Biosignalanalysis, University of Muenster, Germany; h Athinoula A. Martinos Center for Biomedical Imaging, Massachusetts General Hospital, Charlestown, MA, USA; i Harvard Medical School, Boston, MA, USA; j NatMEG, Department of Clinical Neuroscience, Karolinska Institutet, Stockholm, Sweden; k Centre for Human Brain Health, University of Birmingham, Birmingham, UK; l Department of Integrative Physiology, National Institute of Physiological Sciences, Okazaki, Japan; m Department of Functional Neurology and Epileptology, Neurological Hospital & University of Lyon, Lyon, France; n Department of Epileptology, Tohoku University, Sendai, Japan; o Department of Psychological and Brain Sciences, Indiana University, Bloomington, IN, USA; p Department of Neuroscience, Imaging and Clinical Sciences, Università degli Studi G. D'Annunzio, Chieti, Italy; q Institute of Clinical Neuroscience and Medical Psychology, and Department of Neurology, Heinrich-Heine-University, Düsseldorf, Germany; r Institute for Learning & Brain Sciences, University of Washington, Seattle, WA, USA; s Department of Physics, University of Washington, Seattle, WA, USA

**Keywords:** AEF, auditory evoked field, BOLD, blood-level oxygen dependent, CKC, corticokinematic coherence, CMC, cortex–muscle coherence, DCM, dynamic causal modeling, EEG, electroencephalography, ECD, equivalent current dipole, ECoG, electrocorticography, fMRI, functional magnetic resonance imaging, HE, hepatic encephalopathy, IAP, intracarotid amobarbital procedure, ICA, independent component analysis, IES, intracutaneous epidermal electrical stimulation, ISI, interstimulus interval, MEG, magnetoencephalography, MNE, minimum norm estimate, MRI, magnetic resonance imaging, MUSIC, multiple signal classification, SEF, somatosensory evoked field, SNR, signal-to-noise ratio, SQUID, superconducting quantum interference device, SSS, signal-space separation, STN, subthalamic nucleus, TMS, transcranial magnetic stimulation, tSSS, temporo-spatial signal space separation, VEF, visual evoked field, Magnetoencephalography, Electroencephalography, Clinical neurophysiology, Evoked and event-related responses, Transient and steady-state responses, Spontaneous brain activity, Neural oscillations, Analysis and interpretation, Artifacts, Source modeling, Epilepsy, Preoperative evaluation, Stroke, Pain, Traumatic brain injury, Parkinson’s disease, Hepatic encephalopathy, Alzheimer’s disease and dementia, Neuropsychiatric disorders, Brain maturation and development, Dyslexia, Guidelines

## Abstract

Magnetoencephalography (MEG) records weak magnetic fields outside the human head and thereby provides millisecond-accurate information about neuronal currents supporting human brain function. MEG and electroencephalography (EEG) are closely related complementary methods and should be interpreted together whenever possible.

This manuscript covers the basic physical and physiological principles of MEG and discusses the main aspects of state-of-the-art MEG data analysis. We provide guidelines for best practices of patient preparation, stimulus presentation, MEG data collection and analysis, as well as for MEG interpretation in routine clinical examinations.

In 2017, about 200 whole-scalp MEG devices were in operation worldwide, many of them located in clinical environments. Yet, the established clinical indications for MEG examinations remain few, mainly restricted to the diagnostics of epilepsy and to preoperative functional evaluation of neurosurgical patients. We are confident that the extensive ongoing basic MEG research indicates potential for the evaluation of neurological and psychiatric syndromes, developmental disorders, and the integrity of cortical brain networks after stroke. Basic and clinical research is, thus, paving way for new clinical applications to be identified by an increasing number of practitioners of MEG.

## Highlights

• The main principles of magnetoencephalography (MEG) and the value of combined MEG and EEG are discussed.
• Established and some potential future clinical applications of MEG are reviewed.
• Practical guidelines for clinical MEG examinations are presented.

## 1 Background

### 1.1 General

These are the first IFCN-endorsed clinical guidelines for magnetoencephalography (MEG). MEG guidelines have been previously published by the American Clinical Magnetoencephalography Society (Bagic et al., 2009, Burgess et al., 2011, Bagic et al., 2017), the Japanese clinical MEG community (Hashimoto et al., 2004), and the MEG research community (Gross et al., 2013a).

MEG has existed for close to 50 years and is currently used as a clinical tool for assessing human brain function. The first human scalp EEG recordings, published about 90 years ago (Berger, 1929), were of spontaneous activity in both healthy subjects and patients. During the 1960s, with the introduction of laboratory computers, evoked-potential recordings and quantitative methods became widely available in the EEG community but still the main clinical use of EEG relied on interpretation of spontaneous activity. In contrast, soon after the first demonstrations of the detection of the magnetic counterpart of the alpha rhythm, systematic MEG recordings began with evoked-response recordings, for which an adequate signal-to-noise ratio (SNR) was obtained by signal averaging. This approach also allowed mapping the entire MEG pattern by moving the single-channel MEG sensor from one position to another between repeated measurements. However, clinically relevant and reliable recordings of spontaneous MEG had to wait for the introduction of multichannel instruments covering the whole scalp. The tiny size of neuromagnetic fields makes MEG recordings technically challenging, and in addition to low-noise sensors, special care has to be paid to elimination of artifacts that can easily contaminate the recordings. Note, however, that MEG may be less sensitive than EEG to muscle artifacts (Claus et al., 2012, Muthukumaraswamy, 2013). Overall, MEG and EEG complement each other as will be described below.

The temporal resolutions of MEG and EEG are identical—in the millisecond range—but MEG offers a number of advantages over scalp EEG recordings. Skull and scalp smear EEG potentials but do not affect magnetic fields. Consequently, little information about *in vivo* electrical conductivities of head tissues is required for determining the sources of MEG signals. Therefore, the locations and time courses of the underlying neuronal generators can be inferred more accurately and less ambiguously from MEG than scalp EEG data. The interpretation of EEG recordings is further complicated by the requirement of a reference electrode, whereas no comparable reference site is needed for MEG. The two methods are also differentially sensitive to the orientations of currents, as will be described below.

Several review articles and text books are available for MEG methods and applications (Sato, 1990, Hämäläinen et al., 1993, Del Gratta et al., 1999, Baillet et al., 2001, Hämäläinen and Hari, 2002, Salmelin, 2007, Aine, 2010, Hansen et al., 2010, Hari et al., 2010, Hari and Salmelin, 2012, Pizzella et al., 2014, Baillet, 2017, Hari and Puce, 2017, Hari, 2018). Here, we focus on clinical applications and related research, starting with a review of the basics of MEG physics and physiology.

### 1.2 Basic physiology and physics of MEG

Moving charges form electric currents that generate magnetic fields. How well these fields can be detected at a distance with MEG sensors depends on the spatial configuration of the currents and on the electrical conductivities of different tissues in the head. The basic mechanisms of MEG and EEG generation are discussed in detail, e.g., in a recent primer (Hari and Puce, 2017).

**Fig. 1:**
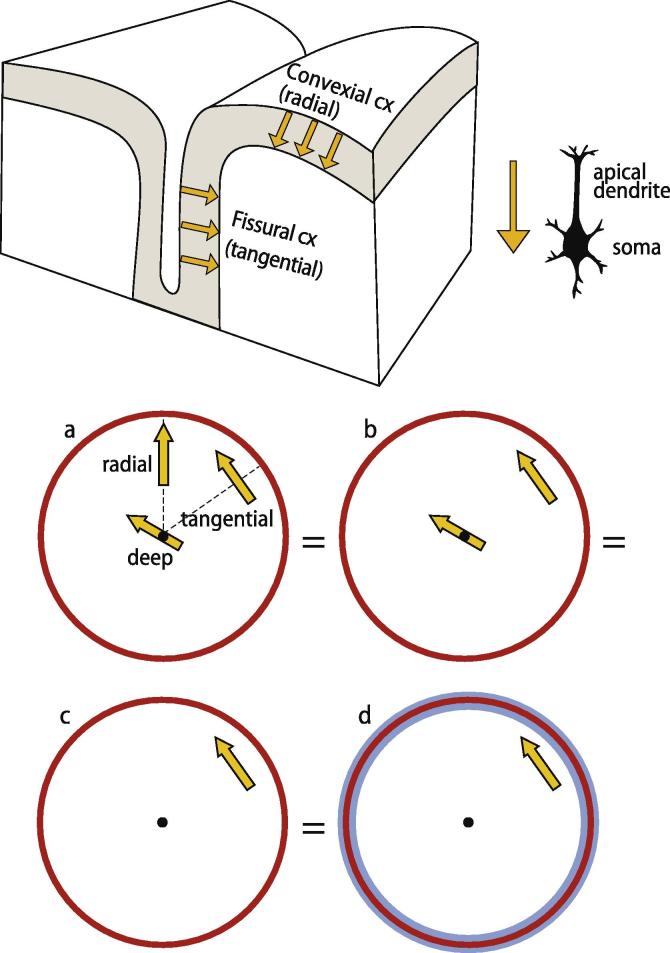
Top: Schematic presentation of convexial and fissural currents in a slab of cortex. The main axis of pyramidal neurons, which are considered to be the main sources of the MEG signals, is perpendicular with respect to the cortical surface. Thus, currents in the walls of fissures are tangential with respect to skull surface and, therefore, are the main contributors of MEG signals. The current direction as such depends on the activation type (excitation, inhibition) of the neuron and the site (superficial, deep) of activation. For more details, see, e.g., Hari and Puce (2017). Modified from Hari and Puce (2017) with the permission of Oxford University Press. Bottom: Currents in the brain and “brain in a nutshell”. Panel (a) shows all possible current orientations in a sphere. The tangential source produces a magnetic field outside the sphere (corresponding to the MEG signals) and is the same as in panels (b–d) exactly because radial currents do not produce external magnetic fields (and as any current in the middle of the sphere is radial). Moreover, concentric inhomogeneities, as in (d) do not dampen nor smear the magnetic field. In other words, all situations (a–d) are equal from MEG's point of view. Modified from Hari and Puce (2017) with the permission of Oxford University Press; the original figure is from Hari et al. (2000).

The main physiological sources of MEG and EEG signals are post-synaptic currents in cortical pyramidal cells. Because the apical dendrites of the pyramidal cells are consistently oriented perpendicular (normal) to the cortical surface, they guide the net macroscopic neural currents to flow perpendicular to the cortical surface (see Fig. 1, top panel).

It is easiest to understand the relationship between cerebral currents and the resulting MEG signals with focal models of current flow (current dipoles) within a spherical volume conductor (see Fig. 1, bottom panel). MEG is most sensitive to cortical currents that are oriented tangential to the skull, that is, perpendicular to the walls of cortical fissures (Fig. 1 top panel). If the current is tilted with respect to the skull surface, its tangential component can produce a strong MEG signal, especially if the current is located in cortical regions close to the skull (Hillebrand and Barnes, 2002). Despite MEG's preference to superficial currents, both recorded data and modelling imply that MEG can see also deeper activity (Attal et al., 2009, Coffey et al., 2016). Instead, EEG is sensitive to signals from both gyral and convexial cortex (Fig. 1 top panel), and it is more sensitive than MEG to deeper brain structures (Hari, 1990, Hari and Puce, 2017). Altogether, MEG and EEG complement each other, and the best non-invasive electrophysiological access to brain function is obtained when both signals are measured and interpreted together.

With the introduction of whole-scalp MEG systems in 1990s, it became possible to record the magnetic field pattern outside the head, instead of performing a serial mapping—often over several days—using a single sensor or a small sensor array. The effects of fluctuating vigilance and cognitive states between measurements were thus eliminated. It also became possible to record brain rhythms and their reactivity during various tasks and in response to different stimuli, and to probe the brain mechanisms of cognition, including speech production, perception, and social interaction.

### 1.3 Overview of MEG signals

#### 1.3.1 Spontaneous activity

Brain rhythms measured with MEG have distinct dominant frequencies (similar to those in EEG), as well as characteristic spatial patterns that can typically be differentiated more clearly with MEG than with EEG (Niso et al., 2016). These rhythms vary as a function of the subject’s behavior, attention, mental state, and vigilance. Importantly, changes in the frequency content and rhythmicity of the spontaneous MEG (and of EEG) “background activity” can indicate various types of brain abnormalities.

The studies of brain’s spontaneous rhythmic activity experienced a renaissance in the 1990s when whole-scalp MEG devices became available and cerebral sources of various brain rhythms, especially in a frequency range from 1 to 40 Hz (Hari and Salmelin, 1997), could be identified in specific brain areas. Below we briefly discuss these rhythms, but refer the reader to reviews and textbooks for more details.

The parieto-occipital alpha rhythm has generators widely spread in the posterior brain with two main source regions: in the parieto-occipital sulcus and the calcarine sulcus (Lü et al., 1992, Salmelin and Hari, 1994b, Hari et al., 1997, Jensen and Vanni, 2002, Manshanden et al., 2002, Keitel and Gross, 2016). Importantly, the source configuration can vary even during a single alpha spindle of less than a second in duration (Salmelin and Hari, 1994b).

As expected, the reactivity is similar for MEG and EEG alpha rhythms: the parieto-occipital alpha rhythm is typically present during eye closure and suppressed with eye opening. However, even in the eyes-open condition, prominent alpha can occur if the subjects are drowsy, bored or cannot fixate their gaze, or are engaged in a demanding task that does not require visual input. The peak frequency of the alpha rhythm changes across the lifespan, gradually increasing in childhood to adult levels, then decreasing in senescence (Pearl et al., 2018).

In general, brain rhythms with alpha-range frequencies reflect decreased excitability of specific brain regions. Note that the large amplitude of the rhythm does not necessarily imply stronger activity, but rather increased synchrony of the engaged neurons. Parieto-occipital alpha power both during rest and during working-memory tasks is thought to reflect inhibition of visual regions, serving to reduce the interference from visual input, which might disturb working memory retention (Jensen et al., 2002, Klimesch et al., 2007, Scheeringa et al., 2009, Payne and Sekuler, 2014).

The time course of mu rhythm has a typical arched shape because it is comprised of two main components, one around 10 Hz (sometimes called the “alpha” band) and another around 20 Hz (sometimes named as the “beta” band). The latter is dominant in precentral motor cortex, whereas the former occurs slightly more posteriorly and has been linked to somatosensory function (Salmelin and Hari, 1994a). The 20-Hz component of the mu rhythm provides a reliable tool to monitor the functional state of the primary motor cortex. Specifically, 20-Hz suppression begins 0.5–2 s prior to a voluntary movement, with a post-movement rebound typically peaking about 0.5 s after the movement ends. This type of mu suppression can also occur during action viewing and motor imagery (Schnitzler et al., 1997, Hari et al., 1998). Similar to the posterior alpha rhythm, the nature of the mu rhythm can be aptly assessed using power-spectral methods that can distinguish the two frequency components of the mu rhythm. The presence of the 20-Hz component of the Rolandic rhythm likely reflects inhibition of the primary motor cortex (Chen et al., 1999), for example during immobility.

Direct recordings from the human subthalamic nucleus (STN) have shown discernable beta-range activity (Brown et al., 2001). A study combining MEG and direct STN recordings demonstrated coherence (see later) between beta-band signals in primary motor cortex and STN (Hirschmann et al., 2011), suggesting frequency-specific coupling between these two brain areas. GABAergic neurons are involved in the generation of beta and gamma rhythms. For example, the GABA-agonist benzodiazepine increases the motor-cortex beta power (Jensen et al., 2005) and decreases its frequency. In the clinical environment, accentuated beta rhythms are frequently seen in patients who use benzodiazepines or barbiturates, and the typical frontal predominance of the EEG beta can be explained by generators in the motor cortex (Jensen et al., 2005).

In general, beta rhythms (14–30 Hz), as elicited in sensorimotor and cognitive tasks, are suggested to maintain the “*status quo*” in local brain regions (Engel and Fries, 2010) although alternate explanations have been suggested recently (Spitzer and Haegens, 2017).

Higher-frequency activity (>30 Hz) can occur in at least six distinct “gamma” frequency bands extending up to 200–600 Hz (Uhlhaas et al., 2011) and originating in different parts of the brain (Hoogenboom et al., 2006). EEG gamma activity can be contaminated by muscle artifacts and microsaccades (Yuval-Greenberg et al., 2008), and the muscle activity contamination is more severe in EEG than in MEG recordings (Claus et al., 2012). The gamma-band activity as such can be detected reliably with both EEG and MEG (Muthukumaraswamy and Singh, 2013).

A large literature of intracranial EEG in patients, and scalp EEG and MEG recordings in healthy subjects documents both facilitatory and suppressive roles for gamma oscillations in perception and cognition (Fries et al., 2007, Jensen et al., 2007). The apparent ambiguity of such findings is due, in part, to the different types of cortical circuits, where both top-down or bottom-up gamma activity could be either excitatory or inhibitory (Sedley and Cunningham, 2013). The large variability of findings and the difficulty to separate gamma activity from artifacts caused by muscular activity (Muthukumaraswamy, 2013) and microsaccades (Yuval-Greenberg et al., 2008) means that great care must be taken when using MEG and EEG gamma-range rhythms in clinical studies. Nevertheless, important advances have been made in associating gamma-band oscillations and psychiatric disorders (Uhlhaas and Singer, 2010, Uhlhaas and Singer, 2012).

In clinical EEG, the theta (4–7 Hz) and delta (≤3 Hz) rhythms have been associated with lowered vigilance and brain pathology (Schomer and Lopes da Silva, 2018). Moreover, delta activity is prominent in the deeper stages of sleep, and changes in theta rhythms have been associated with cognitive functions, e.g., encoding/retrieval of spatial information from episodic memory and working-memory maintenance (Hasselmo and Stern, 2014, Hsieh and Ranganath, 2014). While many of these latter observations are based on findings in the rat, recent MEG work points to the importance of theta-band activity for human memory (Staudigl and Hanslmayr, 2013). Furthermore, the amplitude of gamma bursts varies with the phase of the theta or that of other slower activity (up to alpha) (Canolty and Knight, 2010, Colgin, 2013, Florin and Baillet, 2015).

To avoid confusion, one should always specify the frequency and generation site of a rhythm. The term “alpha activity” would be best limited to the posterior parieto-occipital alpha rhythm. Unfortunately, the very unspecific term “alpha” is often used when discussing the 10-Hz component of the sensorimotor mu rhythm as well as activity in this frequency band generated elsewhere. Additionally, in children where cortical rhythms often occur in different frequencies than in adults, posterior rhythms corresponding to the posterior adult alpha rhythm can be in the adult theta range.

#### 1.3.2 Evoked responses

Any abrupt or strongly-modulated sensory stimuli can elicit strong onset responses. Both MEG and EEG responses are affected by stimulus parameters, including repetition rates, and variables such as the subject’s vigilance, motivation, height, and age. Thus, clinical recordings should be made in standardized conditions, and normative values for evoked-response amplitudes and latencies should be available from each laboratory. Source locations and strengths as a function of time should also be documented whenever possible.

Sensory stimuli can elicit both *evoked* and *induced* activity: evoked signals are time and phase-locked to the stimulus (onset) whereas the induced signals are not; together they form the *total activity* elicited by the stimulus. Evoked responses are typically visualized by averaging responses to individual stimuli, time-locked to stimulus onsets.

If the individual responses are identical and the noise is normally distributed, the SNR of the averaged signals (the signal amplitude divided by the standard deviation of the noise) increases proportional to the square root of the number of averaged responses or trials (Hari et al., 1988). The induced activity that is not consistently time- and/or phase-locked to stimulus onset is severely attenuated by time-locked averaging. However, it can be detected by computing the power (or rectified amplitude) of the signal as a function of time in selected frequency bands. The induced activity is also visible in time–frequency representations (Tallon-Baudry and Bertrand, 1999).

Because evoked-response amplitudes decrease with shortening interstimulus interval (ISI), it is possible to find an optimum ISI for the best SNR per a given measurement time as has been shown for example for responses to painful (Raij et al., 2003) and proprioceptive (Smeds et al., 2017) stimuli. Such optimum ISI is useful in clinical recordings to make them as efficient as possible within the time constraints of the examination.

In healthy subjects, the typical waveforms are very similar for evoked fields (MEG) and evoked potentials (EEG), but with some important differences because of the different relative weighting of (multiple) tangential and radial sources seen by these two methods (see Fig. 1). In general, the shorter the latency, the smaller the response, and early responses are more resilient than later responses to stimulus repetition, medication, and vigilance changes. Therefore, the reliable early responses are, despite their relatively small size, commonly utilized in clinical assessment.

## 2 Acquisition and analysis of MEG signals

### 2.1 MEG instrumentation

The challenge for MEG instrumentation is the detection of extremely weak magnetic fields (from 10^−15^ to 10^−11^ tesla, or T) in the presence of a very noisy background generated by external electrical and magnetic equipment (∼10^−7^ T and above). Properly designed hardware and software must, therefore, combine high sensitivity with the ability to reject noise arising from sources outside the brain.

**Fig. 2:**
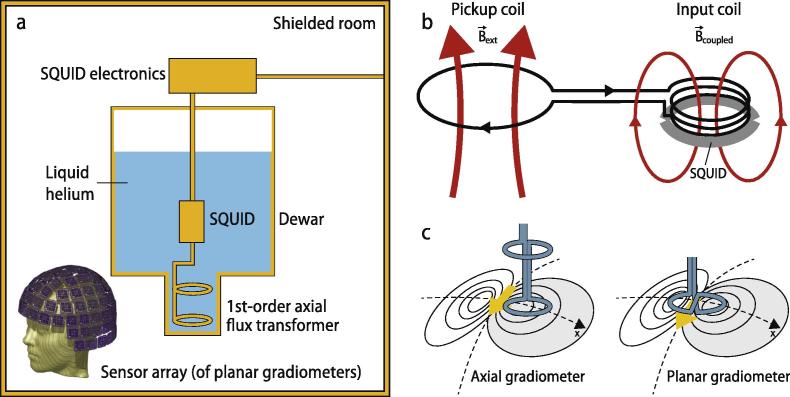
Schematics of MEG instrumentation. (a) A single-channel axial gradiometer and associated SQUID inside a dewar filled with liquid helium. Bottom depicts the sensor array of a 306-channel MEG helmet where each sensor unit contains two orthogonal planar gradiometers and one magnetometer. (b) Flux transformer and SQUID. The external magnetic field generates in the pickup coil (a part of the flux transformer that can take a shape of a magnetometer, or an axial or planar gradiometer) a current that flows in the superconducting loop where one part (input coil) then couples by means of a magnetic field into the SQUID. The electronics monitors the state of the SQUID. Modified from Hari and Puce (2017). (c) Axial and planar gradiometers. An axial gradiometer detects the largest signal a couple of centimeters away from the site of the local source (arrow), whereas the planar gradiometer detects the maximum signal just above the source. Note, however, that the signal in the planar gradiometer depends strongly on its orientation; be it rotated by 90 degrees, the obtained signal would in this case vanish. Thus, devices using planar gradiometers have two orthogonal planar gradiometers at the same sensor unit (see the bottom left insert in (a)). Modified from Hari and Puce (2017) with the permission of Oxford University Press.

The state-of-the-art commercial MEG systems include about 300 magnetic-field sensors in a cryogenic vessel. The main components of such a system (see schematic in Fig. 2a) are (1) the *superconducting quantum interference device (SQUID)* sensors with their related electronics, (2) the *flux transformers* that couple the neuromagnetic field to the SQUIDs, and (3) the cryogenic vessel, the “*dewar*”, containing liquid helium. The characteristics of these components may vary according to specific institutional needs. Additionally, the MEG systems are located inside magnetically (and electrically) shielded rooms to reduce environmental noise to a level compatible with the brain-signal measurements.

#### 2.1.1 Flux transformers

The measured magnetic field is coupled to the SQUIDs with the help of a flux transformer, composed of two coils. The pickup (detection) coil senses the magnetic field of interest while the other coil, the input coil, couples the field to the SQUID (Fig. 2b). It is technically convenient to use a pickup coil that is separate from that of the SQUID loop. Because the flux transformer is a loop made of superconducting wire, the magnetic flux threading it is constant. Therefore, if a magnetic field is applied to the pickup coil, a current proportional to it will arise in the flux transformer. This arrangement differs from the usual case of resistive coils where only the derivative of the field over time induces a current in the loop.

The simplest flux transformer is a *magnetometer* made from a single turn (or few turns) of superconducting wire. However, a magnetometer is sensitive to various artifacts and external noise, which decreases its specificity to brain signals.

More complicated flux-transformer geometries can be used to reduce sensitivity to noise sources but with minor loss of sensitivity for the neuronal sources of interest. The most commonly used flux transformer of this type is the *first-order gradiometer*, made by adding a second coil wound in an opposite sense. The two coils may be displaced along the normal of the coil plane, producing an axial gradiometer (Fig. 2c, left bottom panel), or along the coil plane, producing a planar gradiometer (Fig. 2c, right bottom panel). The magnetic field sensed by this type of a coil is, therefore, the difference of the average fields sensed by the two coils. Planar gradiometers have the benefit that they can be fabricated using thin-film techniques. Usually, the SQUID itself is located on a separate chip attached to the larger flux transformer. For example, one widely-used configuration involves three detection coils integrated in the same silicon chip, namely two planar gradiometers, along two perpendicular axes, and one magnetometer. An additional benefit of a planar gradiometer is that it detects the highest signal directly above the cortical sources (see Fig. 2).

#### 2.1.2 SQUIDs

Modern MEG instrumentation employs SQUIDs to detect magnetic fields of the order of 10^−15^ T. The basic principles of SQUID rely on the properties of a small superconducting loop interrupted by two weak links (Josephson junctions). A wide recording bandwidth (≥10 kHz) is provided together with a flat noise spectrum above approximately 1 Hz. Consequently, SQUIDs are suitable for the detection of brain’s magnetic fields ranging from DC [that is, 0 Hz] to 1000 Hz and above. Effectively, the SQUID with its electronics acts as an extremely low-noise magnetic-flux-to-voltage converter. Detailed technical information can be found in reviews on SQUID sensors and on biomagnetic instrumentation (see for example, Del Gratta et al., 2001, Körber et al., 2016).

#### 2.1.3 Dewar

The dewar is a critical part of the MEG instrument (Fig. 2a) and must satisfy several requirements, including the following: (1) The distance of the detection coils from the head of the subject must be as small as possible, since the field intensity decreases at least as 1/r^2^, where r is a distance between the source and the detector. (2) The magnetic noise should be less than, or at least comparable to the noise of the sensors. (3) The volume of the dewar must be large enough and the boil-off small to make the refill interval feasible for practical operation. The dewar is typically made of fiberglass with a vacuum space between inner and outer shells to eliminate heat transfer by conduction. To avoid heating through radiation, multiple layers of superinsulation (mylar with an aluminum coating on one side) are wrapped around the inner portion of the dewar to provide shielding and to keep the system cool as long as possible. However, thermal currents can flow on the aluminum-covered side of the mylar and thereby increase magnetic noise of the dewar. Commercial biomagnetic dewars exhibit noise figures below 10^−15^ T/Hz^1/2^. A dewar’s helium capacity of 50–70 liters requires a helium refill every 5–9 days. Weekly refill intervals are preferred in the clinical environment because refills can then be more easily scheduled at the same time each week. Recently, closed-cycle cryocoolers have been introduced for helium recycling, which represents a major breakthrough that decreases helium costs and environmental burden and enables successful long-term MEG operation without helium refills (Körber et al., 2016, Okada et al., 2016, Wang et al., 2016).

#### 2.1.4 Shielded room

Magnetically shielded rooms are relatively large, with typical inner dimensions of 3 × 4 × 2.5 m^3^, and they thus provide a comfortable environment for the patient. They typically comprise eddy-current shielding by layers of metals with high conductivity (copper or aluminum) and magnetic shielding by layers of high-magnetic-permeability (iron–nickel) alloys. Typical medium-quality shielded rooms are built using two layers of high-permeability materials and a thick layer of high-conductivity material (usually aluminum). Light-weight rooms, with smaller amount of mu-metal, combined with active shielding, are also available (Taulu et al., 2014).

#### 2.1.5 Future developments of instrumentation

The advent of novel magnetic sensor technologies has led to new developments in MEG instrumentation. High critical temperature (high-T_c_) SQUIDs are currently being tested in small- and middle-sized multichannel systems (Öisjöen et al., 2012, Körber et al., 2016). The major advantage of the high-T_c_ SQUIDs is that they can be operated at liquid nitrogen temperature (∼77 K), thus requiring much less complex dewar construction. Moreover, they can be placed closer to the brain than the low-temperature SQUIDs, thereby providing better spatial resolution (Iivanainen et al., 2017), as long as their higher noise does not compromise this advantage.

Optically pumped magnetometers (OPMs) (Kominis et al., 2003) have also been introduced for brain recordings although their use in large multichannel instruments is still under exploration (Boto et al., 2017; Boto et al., 2018). OPMs are less sensitive than the traditional SQUIDs, but because they can be positioned directly on the scalp and thereby closer to the neural sources, the measured signals will be larger and higher spatial frequencies can be sampled. Importantly, the OPMs operate in room temperature and have a relatively small footprint. There is thus the prospect that such systems could one-day become easily movable and adaptable to different head sizes. Finally, a new generation of superconducting sensors, namely hybrid quantum interference devices (HyQUIDs), has been recently developed (Shelly et al., 2016).

Instrumentation employing any of the above new technologies should result in lower fabrication and operating costs, and thus could spread the use of MEG systems more widely to clinical environments. Several well-known MEG signals, such as the spontaneous alpha rhythm and auditory and somatosensory evoked fields, have been used as physiological test signals demonstrating the feasibility of these new devices (Borna et al., 2017, Boto et al., 2017).

Additional technological developments aim to mitigate problems related to head movements. One possibility is to immobilize the patient’s head during an MEG recording by means of individualized head casts constructed from foam resin in the shape of the scalp surface obtained from the patient's structural MRI and the inner surface of the dewar. These casts fitting and fixing the patient's head to the dewar can greatly reduce head-motion artifacts (Meyer et al., 2017); importantly, the head can be repositioned identically on multiple occasions during follow-up studies. Current technology also allows the head position and orientation with respect to the fixed sensor array to be measured several times per second so that movements can be corrected for in the subsequent analysis (Uutela et al., 2001, Taulu et al., 2005). Moreover, the hybrid MEG–MRI device where MEG and ultralow-field structural MRI can be recorded in the same session provides accurate coregistration of anatomical (MRI) and functional (MEG) information (Vesanen et al., 2013).

### 2.2 General aspects of MEG analysis

As with EEG, it is important to start the analysis with visual examination to assess data quality. In general, the pre-processing and other analyses of MEG signals, except source analysis, are very similar to those for EEG. We refer the reader to published guidelines for reporting MEG data (Gross et al., 2013a). One important strength of MEG is that it can often identify several separate source areas activated sequentially both during normal cognition (Hari et al., 1993a, Nishitani and Hari, 2000, Nishitani and Hari, 2002) and during epileptic discharges.

Because clinical decisions have to be based on the data of an individual patient, with a comparison with normative values, one should not rely too much on automated analysis techniques before their reliability and reproducibility have been clearly demonstrated. Currently, it is preferable to use semiautomatic procedures with operator intervention to check intermediate results between analysis steps to ensure quality control in data analysis.

Spontaneous activity in MEG (as well as EEG) can be quantified by means of power spectra, or by using a wavelet-based time-frequency analysis that displays the frequency changes as a function of time, for example around events of interest. Active sources can be determined either by fitting current dipoles to several peaks of narrowly-filtered cycles of a brain rhythm (one data point per cycle) and then examining the cluster’s centroid and spatial extension, or by using distributed source-estimation methods to reconstruct the distributions of sources in 3D across the brain or on the cortical surface (Baillet et al., 2001).

### 2.3 Data filtering and sampling

If the signals of interest and noise occur in different frequency bands, filtering (high-pass, low-pass, band-pass, or notch) is an effective method to improve the SNR as some frequency bands of the measured signals are eliminated or suppressed.

The general principles of filtering are the same for MEG and EEG. For example, the Nyquist sampling criterion should be followed, meaning that the sampling frequency has to be at least two times the highest frequency of interest in the data. This criterion is normally enforced by the commercial MEG systems. In subsequent processing, digital filters will be employed. Since digital filters can be non-causal, the filter properties should be understood and scrutinized in the physiological interpretation of the data (Ramkumar et al., 2013). Moreover, filtering of finite-length temporal signals can produce “ringing” due to edge effects, and thus it is generally recommended to apply filters on continuous rather than epoched data. Ringing can also occur if the filter is too narrow.

Notch filters can be useful against artifacts containing a narrow set of frequencies, such as power-line interference (50 Hz or 60 Hz depending on the country) and its harmonics. That said, the filtering can be problematic if the signal of interest falls within the same frequency range as the power-line interference.

When relative timing of brain responses, with respect to stimulus or another brain event, is of high interest, special attention should be paid to the properties of the applied digital filter. Such timing requirements are common in MEG studies. For example, zero-phase lag filters should be used when averaging spikes, whereas causal filters are preferred when sources related to the onset portions of the averaged spikes are constructed. This distinction is necessary because a causal filter ensures that the filtered signal at the time point of interest is only affected by the activity at that particular time and at previous time points. A signal that is processed with a zero-phase filter, which is non-causal, would also be affected by future time points (Jackson, 1996, Oppenheim and Schafer, 2009, Widmann et al., 2015).

### 2.4 Artifacts

MEG signals are smaller than many biological and non-biological magnetic fields, and thus prevention and recognition of artifacts is an important consideration in an MEG recording. It is always preferable to prevent unwanted non-brain signals during data collection rather than to attempt to correct or compensate for them during data analysis.

To detect potential artifacts related to instrumentation (noise in SQUIDs, line-frequency contamination, slow drifts), the performance of the MEG system should be checked regularly (at least once a month) with a phantom that contains current sources with known geometry and temporal patterns of activation.

The main procedures to record clean data are (1) to prevent artifacts from occurring in the first place, (2) to reject MEG (and any simultaneously recorded biosignal) epochs grossly contaminated by artifacts, and (3) to correct or remove the remaining artifacts by post-processing. These basic procedures have been recently summarized by Hari and Puce (2017). It is quintessential to learn the generation mechanisms and the distributions of the most typical artifacts so that the artifacts can be monitored and already noted during data collection. For example, slow signal shifts may indicate that magnetic material in the clothing is moving with respiration. Clear instructions to the patient before the recording may help to avoid eye-movement, eye-blink and muscle-related artifacts. The waveforms of these artifacts are similar to those in EEG recordings and, thus, quite easy to recognize if the operator has EEG experience.

Non-physiological artifacts can arise from sources inside (e.g., implanted stimulators) or outside the patient's body (e.g., clothing, stimulation and recording equipment), or even outside the laboratory. Patients may have therapeutic instrumentation that cannot be removed for the duration of the MEG recording, and in these cases efficient post-processing of the data is necessary.

Consequently, the MEG recordings contain, in addition to the signals of interest, various environmental and patient-related artifacts. Some artifacts arise outside or even far away of the measurement array, e.g., from moving elevators elsewhere in the building, while some are much closer (e.g., dental braces), even in the sensor array itself producing uncorrelated sensor noise.

Patient movements can produce large low-frequency fluctuations and/or high-frequency muscular artifacts, but even without such contamination, the estimated source locations will contain errors if the head has moved during the recording. Continuous head-movement tracking, followed by application of a device-independent signal decomposition algorithm, can help to compensate for head movements (Larson and Taulu, 2017b) and thereby improve the accuracy of source estimation.

Some MEG devices have reference sensors located far from the head, essentially recording external interference with very little contribution from the brain. With the help of these reference sensors one actually forms long-baseline “software gradiometers”, which can effectively suppress artifacts arising in the environment.

Signal space projection (SSP) (Uusitalo and Ilmoniemi, 1997) can be used to suppress external magnetic fields. SSP usually employs an “empty-room” recording lasting for a few minutes and conducted without the subject but otherwise identically (with the same recording and stimulation equipment) as the clinical MEG investigation itself. SSP is useful in rejecting or decreasing signal contamination from eye blinks and heartbeats, as well as from distant external noise sources, such as elevators in the building or moving vehicles. This procedure works because the artifacts can be well represented as a weighted sum of the principal signal patterns that the “empty-room” data tend to characterize, even if the distant artifact sources change with time. SSP usually affects the brain signals to some extent as well. Therefore, the subsequent analysis has to take into account the use of SSP and apply appropriate correction to the forward model for the source estimates to be correct.

The signal space separation (SSS) is an alternative method for artifact reduction for data collected with modern MEG systems that contain over 200 channels and, therefore, oversample the detectable MEG field patterns (Taulu et al., 2005). SSS relies on a physics-based spatial filter that is determined computationally. It differs from SSP, which uses a filter optimized ion the basis of a noise measurement. Inherent to SSS is a signal reconstruction step that usually allows the source estimation to proceed without explicit knowledge of the applied spatial filter.

**Fig. 3:**
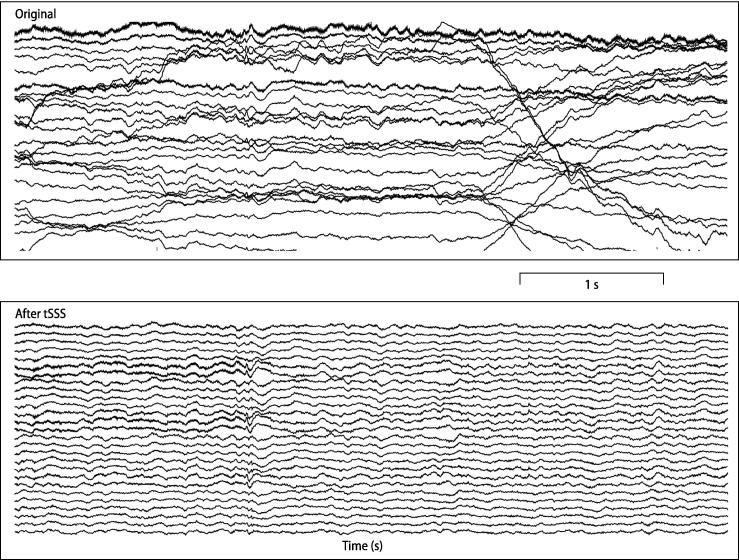
Effect of tSSS cleaning of slow artifacts caused by small residual magnetized particles left from skull drilling. Spontaneous MEG data were recorded with a CTF-275 device in an epileptic patient who underwent craniotomy and a temporal resection. Top panel: Original data. MEG signals from 27 channels are displayed. Bottom panel: tSSS-cleaned data. Filters correspond to the standard CTF data acquisition system with frequency band acquired from DC to 240 Hz. No additional filtering was performed. All traces are from first-order axial gradiometers with 5 cm baseline. Reference-channel information was not applied in these data. Data courtesy of Eliane Kobayashi (McGill University, Montreal, Canada).

Artifact sources close (<50 cm) to the sensor array, such as eyes and head muscles, produce spatially complex field patterns that SSS cannot suppress. In this case, the temporal extension of SSS (tSSS) can be employed (Taulu and Simola, 2006, Taulu and Hari, 2009). The tSSS method generally works with relatively little user intervention, and it is routinely used in clinical MEG investigations involving deep-brain stimulation (DBS) or vagal nerve stimulation (VNS), which both produce strong and complex MEG artifacts (Kakisaka et al., 2013, Airaksinen et al., 2015). Fig. 3 illustrates how tSSS cleans spontaneous MEG data recorded from an epilepsy patient in whom magnetic particles in the skull produced large-amplitude drifts to the recordings.

Independent component analysis (ICA) is a useful method to extract artifacts from the collected data on the basis of their statistical properties (Mantini et al., 2011, Hari and Puce, 2017). The downside of ICA is that the waveforms must be visually inspected and interpreted as artifacts or non-artifacts in both space and time, although recent approaches have introduced quantitative measures to identify specific artifacts (Chaumon et al., 2015).

Uncorrelated sensor noise can be suppressed by cross-validation methods. Recently, a comprehensive mathematical framework has been developed that allows optimization of the sensor-noise suppression by fully exploiting both the spatial and temporal properties of the MEG data (de Cheveigne and Simon, 2008, Larson and Taulu, 2017b)

Below we recommend some artifact suppression methods, suitable for dampening different types of nuisance signals. Independent of the methods employed, it is advisable to conduct a measurement in the room void of a patient, since it is useful in subsequent analysis and can be used to identify problems if standard approaches fail. Note that ICA can be used to suppress any kind of artifacts, except head movements, but our recommendations below take into account the amount of user intervention required. In clinical work, manual inspection of signal components required by ICA may be troublesome and one cannot rely blindly on automated procedures, at least at the current stage of methodology. Of course, if the basic statistical assumptions of ICA are not met, the results can be erroneous.

(i) *External noise sources* (distance > 50 cm), including for example traffic, elevators, and electronic laboratory instruments, can be suppressed with reference sensors, SSP, SSS, and tSSS.
(ii) *Spatially correlated artifacts* that cannot be represented as external noise sources, including preamplifier drifts, electronically coupled power-line signal, eye blinks, respiration, and movement artifacts caused by magnetized material, can be suppressed with tSSS and removed with ICA. SSP can be used if stationary artifacts are present in the baseline measurement.
(iii) *Uncorrelated sensor noise*, including thermal noise in the SQUID sensors and flux trapping, can be suppressed with cross-validation methods (de Cheveigne and Simon, 2008, Larson and Taulu, 2017a).
(iv) *Head movements* are important to take into account if they are larger than the otherwise expected source-localization error; such movements are typical during seizures but also occur in healthy infants. Here the recommended suppression methods are SSS and tSSS. Minimum-norm-based methods (see below) can be used as well, but a separate algorithm for the suppression of movement-induced artifacts would need to be applied. Continuous tracking of head position is mandatory.

### 2.5 Source estimation

From the very beginning, MEG analysis has emphasized the need to estimate the actual neural sources of the magnetic field, i.e., to work in *source space*, rather than to investigate the recorded signals only (“*sensor space*”), which is still very common in EEG analysis. This source-space approach is easier in MEG than EEG because reasonably accurate source estimation can proceed even without generation of fully accurate forward models. Source estimation has gradually made its way to EEG analyses as well, despite the additional complexity of the forward model needed, reflecting the benefits of data interpretation in terms of brain sources rather than their remote manifestations on the scalp or outside the head.

Solution of the *forward problem*—that is describing how MEG and EEG signals are generated by known sources—opens up the possibility to find an estimate of the primary currents given the MEG measurements and the calculated forward model. However, this so-called *inverse problem* is ill posed because, in principle, an infinite number of current distributions can explain the sensor-space data and the solutions are also sensitive to noise. Moreover, sources may be silent (not visible) in MEG, EEG, or both. Fortunately, however, these issues can be mitigated. Potential current distributions can be restricted by employing anatomically and physiologically meaningful constraints. Noise sensitivity can be reduced using regularization: the exact match between the measured data and those predicted by model is in part sacrificed to make the estimates more robust (Hämäläinen et al., 1993, Baillet et al., 2001).

In principle, all sensors of an MEG device see every (visible) source in the brain, but with different weights, and thus the time-varying signal of any MEG sensor is a linear combination of the activation time courses of all sources. The goal of solving the inverse problem is to produce source estimates that correctly describe the locations and extents of the sources underlying the measured MEG data and yield their unmixed waveforms.

MEG/EEG source-estimation methods can be divided into three categories: (i) parametric source models, (ii) distributed current estimates, and (iii) scanning approaches.

In *parametric modeling*, one commonly assumes that the cortical activity underlying the measurements is sparse, i.e., salient activity occurs only at a small number of cortical sites, and that each active area has a small enough spatial extent to be equivalently accounted for by a point source, an equivalent current dipole (ECD). This time-varying current-dipole model has been developed to great sophistication in the analysis of evoked responses (Scherg, 1990). The dipole models are often used to explain measurements of early sensory responses, but they can be also successfully employed in modeling more complex MEG data (see, e.g., Salmelin and Hari, 1994a, Salmelin et al., 1994, Nishitani et al., 2004).

In *distributed modeling*, the sources are confined to a volume (typically the brain) or a surface (typically the cortex), and among the multiple current distributions capable of explaining the data, one selects a particular one by imposing an additional criterion.

To date, the most successful method of this kind has been the cortically-constrained minimum-norm estimate (MNE) (Dale and Sereno, 1993, Hämäläinen and Ilmoniemi, 1994, Dale et al., 2000), which selects a current distribution with minimum overall power. The MNE is diffuse, usually overestimating the extent of the source, and thus the extent of the solution should not be interpreted too literally. Yet, it has few parameters, and it is relatively immune to noise and head-model approximations (Stenroos and Hauk, 2013).

The MNE belongs to a large family of source estimation techniques that all share the same underlying concept. Much methodological development has occurred in these algorithms (Uutela et al., 1999, Ou et al., 2009, Gramfort et al., 2012, Gramfort et al., 2013b) as well as in related approaches that include prior assumptions about the distribution and interactions of the sources (Friston et al., 2008, Wipf and Nagarajan, 2009).

In the third class of source estimation methods, *a scanning function,* which depends on the measured data, is evaluated at each candidate source location. A high value of the function is taken to indicate a likely source location. Two closely related examples of these types of methods are the linearly-constrained minimum variance beamformer (LCMV, van Veen and Buckley, 1988, Hillebrand et al., 2005, Sekihara and Nagarajan, 2008) and multiple signal classification (MUSIC, Mosher et al., 1992, Mosher and Leahy, 1998).

The beamformer method has gained a lot of popularity among MEG researchers while its use in EEG analysis has been limited, likely because it is quite sensitive to head-modeling errors (Steinstrater et al., 2010). Finally, the scanning approaches differ from the parametric dipole models and distributed models in the sense that the maps they produce are those of statistical scores; importantly, they do not represent current distributions that can explain the measured data.

In general, MEG source localization benefits from accurate volume conductor models. Modern software packages support, with very little user intervention, the use of realistically shaped head models as an alternative to the spherically symmetric head model (Baillet et al., 2011; Gramfort et al., 2013a, Gramfort et al., 2014).

### 2.6 Functional connectivity

MEG can be used to resolve concerted activity of different cortical areas with a fine temporal detail. If each MEG sensor could be uniquely attributed to a specific brain region, estimation of functional connectivity could rely only on an appropriate choice of measures of association between signals. The spread of magnetic fields, however, complicates the problem. For example, even if all brain activity could be equivalently accounted for by a single current dipole, one would measure linearly-related signals on many sensors. For this reason, more realistic and reliable estimates for connectivity between brain areas are generally obtained at the source rather than at the sensor level (Schoffelen and Gross, 2009, Gross et al., 2013a).

It should be remembered, however, that functional connectivity describes related activity between two (or more) brain areas and does not necessarily imply a direct structural connection. For example, a third brain area (C) could drive two other areas (A and B), which can result in high functional connectivity scores between A and B.

The two main approaches to connectivity estimation between neuronal populations are (1) a post-hoc metric of connectivity after some generic and robust source estimation, and (2) the use of an explicit model of connectivity to generate MEG data and hence to estimate connectivity (and causality) as a part of the inversion process.

The most common approach is to first estimate sources without any explicit model of connectivity and then estimate the connectivity post-hoc. The advantage here is that these inversion methods are well understood, general, and not heavily parameterized. The disadvantage is the lack of explicit description of the source connectivity structure. Therefore, one must correct for erroneous apparent connectivity (also termed leakage, field-spread, cross-talk, seed-blur) introduced by the inversion algorithm. As already mentioned, MEG source reconstruction typically relies on recordings that contain a linear combination of data from a finite number (∼300) of MEG sensors. The most-straightforward methods to estimate functional connectivity between two brain regions are those that ignore any coupling that could be due to this linear inversion. For example, one can discard the real (zero-lag) part of the coherence spectrum and only look for signals that are lagged with respect to one another (Sekihara et al., 2011). These lagged time-courses cannot be due to the linear mixing implicit in the inversion (Marzetti et al., 2013). Other approaches strive to linearly regress out any constant coupling terms (Brookes et al., 2012, Hipp et al., 2012, Colclough et al., 2015).

A similar robust (but non-linear) metric is the phase-lag index (Stam et al., 2007, Hillebrand et al., 2012), which tends to zero any zero-lag coupling but is biased away from zero when one narrow-band signal consistently lags, or leads, the other. Making inferences on the causal nature of one brain region on another would again be straightforward if the signals were perfectly known (measured). The complication is that the neuronal current flow at any cortical location is due to the gradual aggregation of post-synaptic potentials/currents over thousands of pyramidal neurons, so that it is difficult to determine the exact onset time of the activity. Moreover, these signals in the two functionally coupled areas may be embedded in different levels of noise, which affect the latency at which the signal is visible.

Granger causality tests the degree to which the prediction of the future of a signal (A) is improved by using the past of another signal (B) in addition to its own. This improvement is taken to indicate a causal connection from B to A. The difficulty here, when dealing with signals that may have differing levels of noise, is that the least noisy signal is generally the best predictor of the future of the other, and the computations easily result in false positives (Nolte et al., 2008). Typically, however, as long as one is aware of these caveats, such methods have been used with success; for example Michalareas et al. (2016) recently showed how MEG measures of causality in gamma and beta bands reflect underlying feedforward and feedback structural connectivity and the hierarchy of 26 visual areas.

Other methods of assessing the flow direction of information in time series include phase-slope index computed across all sensor pairs (Nolte et al., 2008) and measures of directed entropy (Wibral et al., 2013).

Dynamic causal modeling (DCM) constructs an explicit plausible network of biophysically realistic sources that likely generate the MEG data. It typically involves a small number of specified sources and a restricted set of competing hypotheses of connectivity (Kiebel et al., 2009). DCM has the advantage that connectivity (and causality) can be explicitly tested for without concerns about the leakage because there is an explicit model for MEG generation and the generated MEG data are compared with measured MEG data. However, the model typically rests on strong prior hypotheses about the active brain regions and explicit (and complex) biophysical models of how neuronal assemblies interact. Yet, the advantage of DCMs (as they strive to explain all of the measured data) is that new models with different source or connectivity structures can be compared and incrementally improved within the same model-comparison framework (Friston et al., 2007). Most importantly, DCM delivers an explicit framework for testing of effective connectivity, i.e., for causal interactions mediated by both functional and structural connections between brain regions. In this way, for example the time constants and firing rates can be explicitly modeled. The construction of such highly-parameterized models would seem infeasible but can be made tractable within a Bayesian framework in which these many parameters are free to vary within some bounds of mean and precision. The bounds themselves are updated over time to give tractable, biophysically interpretable models that can allow one to make inferences even down to synaptic level (Moran et al., 2011).

Early clinical studies indicate that network behavior is altered in different types of brain disorders (Sanz-Arigita et al., 2010, Olde Dubbelink et al., 2014, Tewarie et al., 2014); however, it is not yet known at this point which measures will be clinically useful.

### 2.7 Correlations between brain and peripheral signals

Human brain-imaging studies aim at exploring interactions between brain and environment: from the environment to the brain (perception) and/or from the brain to the environment (action). Traditionally, such interactions are studied by means of temporal coincidence as in evoked-response studies, where the elicited brain responses are interpreted to reflect the processing of the stimulus.

Any change, be it an external stimulus or a biological signal from the subject herself, can be used as a regressor in the analysis of the MEG (and EEG) data. Useful biological signals include electromyography (EMG), limb acceleration, limb velocity, applied force, fundamental frequency of the voice, electrocardiography (ECG), eye gaze, and even eye blinks.

The analysis typically relies on the application of bivariate measures that quantify statistical dependencies, such as correlation, between the two variables. Practically, cross-correlation is used to account for delays between peripheral and MEG signals. However, often this coupling between the periphery and the brain is present in a specific frequency band, meaning that the analysis methods should be optimized for band-limited interactions. A coherence spectrum (a correlation measure in the frequency domain) quantifies the coupling strength across a range of frequencies. More advanced measures can be used to unravel non-linear dependencies (Quian Quiroga and Panzeri, 2009, Ince et al., 2017), or to quantify the directionality of the coupling (Bastos and Schoffelen, 2015). Coupling can be quantified by using regression techniques to compute impulse–response functions or spectrotemporal receptive fields (VanRullen and Macdonald, 2012, Crosse et al., 2015, Hullett et al., 2016) that characterize the response profile of a specific brain area. Importantly, all these methods are suitable for the analysis of continuous signals, and recordings of a few minutes length can provide sufficient SNR for the identification and quantification of the coupling between periphery and brain.

Several tasks can lead to robust coupling between rhythmic MEG and EMG signals. For example, continuous isometric muscle contraction is associated with coherence at 15–30 Hz between the EMG and the primary motor cortex (Conway et al., 1995, Salenius et al., 1997a, Gross et al., 2000, Salenius and Hari, 2003) or even at 40 Hz (Salenius et al., 1996, Brown et al., 1998).

This cortex–muscle coherence (CMC) originates from oscillatory activity in primary motor cortex that affects the population-level firing pattern of spinal motor neurons (Baker et al., 1999), a likely mechanism for efficient and robust driving of spinal motor neurons both in humans (Schoffelen et al., 2005) and rats (Parkis et al., 2003). The cortex is leading the muscle during isometric contraction (Salenius et al., 1997a, Brown et al., 1998).

The 15–30-Hz cortex-muscle coherence is reduced or abolished after movement onset but can be replaced by coherence at different frequencies, e.g., gamma frequencies around 40–70 Hz (Schoffelen et al., 2005). During slow finger or hand tracking movements, the 6–9-Hz corticospinal coherence becomes manifest as slow amplitude fluctuations in the movement, clearly visible in accelerometer recordings (Gross et al., 2002, Jerbi et al., 2007). Changes in cortex–muscle coherence seem not to be simply a consequence of changes in power of beta rhythms in sensorimotor brain areas, but rather reflect an independent mechanism for efficient motor control in its own right (Gross et al., 2005, Schoffelen et al., 2005, van Wijk et al., 2012). It has been suggested that the cortex–muscle coherence is a manifestation of rhythmic movement control in a cerebello-thalamo-cortical loop (Gross et al., 2002), but more recent studies using corticokinematic coherence (CKC) have demonstrated an important and frequency-specific contribution from the proprioceptive afference during finger and hand movements (Piitulainen et al., 2013, Lim et al., 2014, Bourguignon et al., 2015): In lay terms, the cortex speaks to the muscle at around 20 Hz whereas the muscle replies to the cortex at frequencies below 3 Hz (Bourguignon et al., 2017). CKC allows accurate identification of the primary sensorimotor cortex even in the presence of strong magnetic artifacts (Bourguignon et al., 2016) and it is, thus, more robust than cortex–muscle coherence for patient studies.

**Fig. 4:**
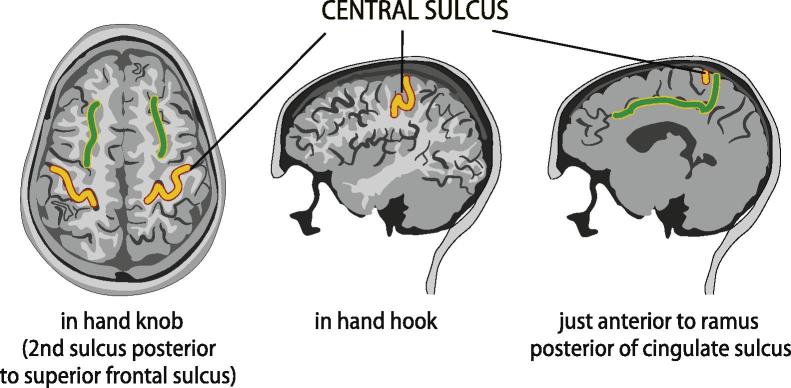
Locating the central sulcus in a structural MRI. A schematic guide to find the central sulcus on the basis of anatomical landmarks in axial (left), parasagittal (middle) and midsagittal (right) sections. The course of the central sulcus is displayed in yellow, and the superior frontal sulci (left) and cingulate sulcus (right) appear in green. This anatomical information should be complemented with MEG information: SEF recordings for pinpointing the somatosensory cortex just posterior to the central sulcus and cortex–muscle coherence recordings to identify the primary motor cortex just anterior to the central sulcus. Adapted from Hari and Puce (2017) with permission from Oxford University Press.

The ability to examine interactions between the motor cortex and spinal cord has potential for clinical applications, although until now the method has only rarely been used at the individual level, except as an additional tool for preoperative identification of the central sulcus (see Fig. 4). Abnormal MEG–muscle coherence has been observed in Parkinsonian patients during withdrawal of levodopa treatment (Salenius et al., 2002), and abnormal EEG–EMG coherence in acute and chronic stroke patients (von Carlowitz-Ghori et al., 2014). In general, cortex–muscle coherence is a pertinent measurement in disorders that are associated with peripheral motor manifestations, such as physiological tremor (Raethjen et al., 2002), essential tremor (Schnitzler et al., 2009), Parkinsonian tremor (Timmermann et al., 2003), and even voluntary tremor (Pollok et al., 2004). These studies have revealed involvement of similar cortical and subcortical motor areas with some distinct group-level differences between types of movement manifestations and disorders (Schnitzler and Gross, 2005). MEG’s advantage over EEG is that it can identify the cortical coherent sources quite accurately.

Several studies have demonstrated robust coupling between quasi-rhythmic auditory and visual speech components (such as phoneme rate, syllable rate, and intonation) and brain activity measured with MEG/EEG (Giraud and Poeppel, 2012, Gross et al., 2013b, Peelle et al., 2013, Crosse et al., 2015). Interestingly, the coupling strength seems to be related to comprehension (Peelle et al., 2013, Park et al., 2015) and to the attentional selection of an individual speech stream in the presence of competing input (Zion Golumbic et al., 2013, Vander Ghinst et al., 2016).

Speech as such is an interesting special case as one can record brain responses to natural speech produced, even online, by another human being. For example, coherence can be detected between an accelerometer signal attached to the throat of the speaker and the MEG signals of the listener (Bourguignon et al., 2013). The speech-entrainment measures allow to investigate deficits of cortical processing in, e.g., dyslexic subjects (Goswami et al., 2014).

### 2.8 Combined use of MEG and EEG

While both MEG and EEG sense postsynaptic currents, they also display clear differences (Hari and Puce, 2017). Source modeling is relatively straightforward for MEG as the effects of the scalp and the skull can be largely ignored (Hämäläinen and Sarvas, 1989, Tarkiainen et al., 2003). Instead, a sufficiently accurate head model must be generated for EEG source analysis, including the distribution of electrical conductivities of head tissues. Most commonly, a three-compartment model has been used to include scalp, skull and brain, but some investigators advocate the inclusion of cerebrospinal fluid into the model to minimize errors (Stenroos and Nummenmaa, 2016). The use of electrical impedance tomography (with scalp EEG electrodes) may ultimately help refine head models for EEG analysis (Dabek et al., 2016). For EEG source modeling, the individual head geometry should be derived from structural MRI data.

EEG signals can be expressed relative to a variety of different reference electrodes (or their combinations), which greatly affects the appearance and often (but erroneously) also the interpretation of data presented in sensor space. Source modeling takes into account the location of the reference electrode. Linked earlobes or mastoids should *not* be used during data collection, because such data cannot be converted in the off-line analysis to correspond to a different single-electrode reference. An average reference, computed across all measurement channels, has been recommended by some authors for modeling high-density EEG data collected with 128 channels or more. For a more detailed discussion and some caveats of this approach, see Hari and Puce (2017).

In MEG analysis, one can avoid many problems of EEG source modeling, for example in infant brains where the relative conductivities of tissues, such as grey and white matter, differ from adult values, the skull has not fully developed to its final thickness, and the fontanels have not yet closed. Thus, EEG signals from a given source at a given distance from an electrode can be stronger than in adults (Azizollahi et al., 2016, Pursiainen et al., 2017). This problem does not exist in MEG.

When MEG and EEG are recorded simultaneously, the fusion of the two data sets provides a more complete picture of the brain’s neural activity (Baillet et al., 1999). For example, the tangential sources could be first characterized using MEG only. The residual in EEG not accounted for by the MEG sources is likely due to radial superficial sources (to which MEG is blind) and to deep sources (which MEG may not be able to record) and could be modeled based on the EEG data (Hari, 1988). Hence, a more complete source model could be specified. Source localization algorithms, which simultaneously consider both EEG and MEG signals together, need to correctly weigh the MEG and EEG signals to avoid one modality biasing the outcome of the joint signal analysis (Baillet et al., 1999). In clinical work, it is useful to carry out EEG and MEG source modeling separately, and then combine the results for clinical interpretation (Ebersole and Ebersole, 2010).

**Fig. 5:**
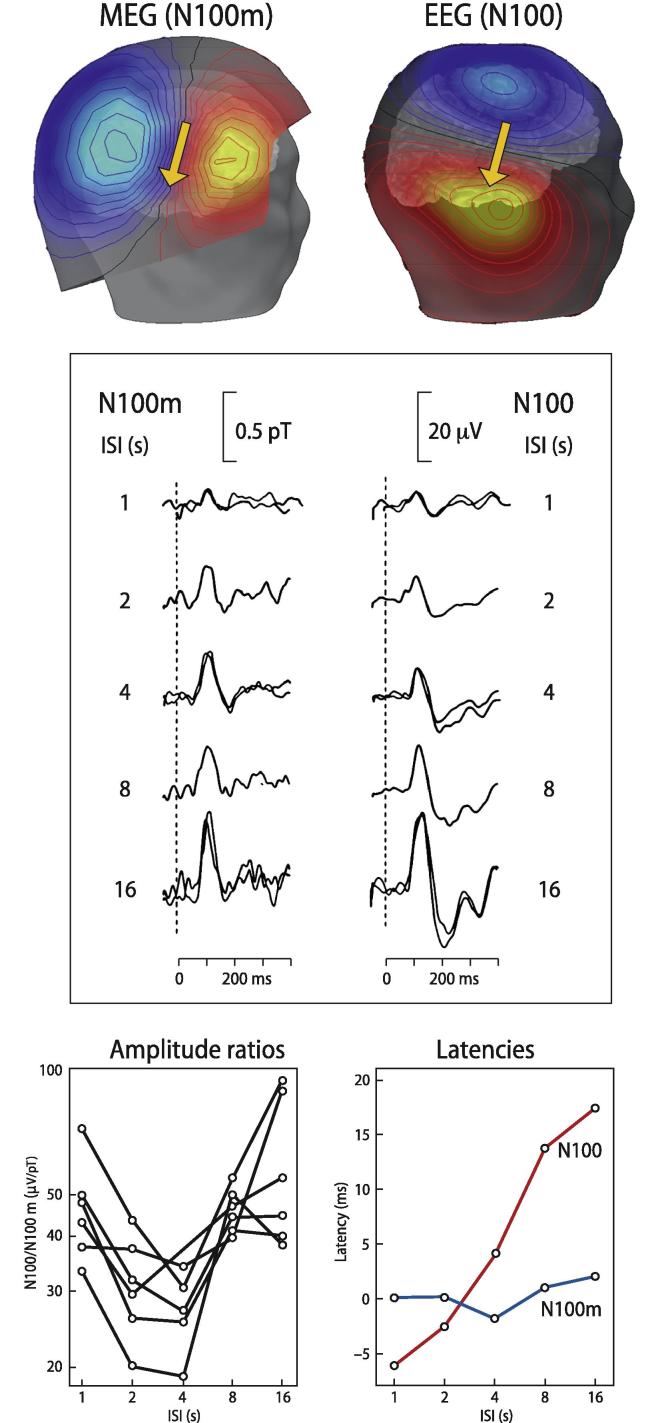
Auditory 100-ms evoked fields and potentials. Top panel: Field patterns for MEG (left, N100m) and EEG (right, N100) responses. These data were simulated for a current-dipole source (arrow) in the auditory cortex. Note that the MEG pattern is displayed about 3 cm above the scalp over the temporal lobe. In future MEG devices where the sensors can be placed very close to the scalp, the MEG field lines will be about 1/3 closer together. The red isocontour lines display magnetic field exiting the head and positive potentials. The blue isocontours depict magnetic fields entering the head and negative potentials. Middle panel: ISI dependence of N100m (recorded with an axial gradiometer from the right posterior maximum of the field pattern) and of N100 (recorded between vertex and right mastoid). Modified from Hari et al. (1982). Bottom left: Ratio of N100/N100m as a function of ISI. Because this relationship is not flat, the electric and magnetic 100-ms responses cannot have identical sources. Bottom right: N100 and N100m latencies as a function of ISI. Latencies also behave differently as a function of the ISI. Modified from Tuomisto et al. (1983).

Combining MEG and EEG data within the same set of experimental manipulations also has the power to differentiate between single or multiple underlying neural sources. A didactic example is the auditory-evoked response peaking about 100 ms after sound onset. In a parametric design that varies inter-stimulus intervals, the magnetic N100m and the electric N100 show both similarities and differences in their behavior (Fig. 5), even though they were originally thought to be the magnetic and electric manifestations of the same neural response. Both N100m and N100 increase in amplitude with progressively increasing inter-stimulus intervals (Fig. 5, middle panel) but with different speeds, which is well reflected in the amplitude ratio of these two signals (Fig. 5, left bottom panel). Because of their different recovery cycles, N100m and N100 cannot be generated by a single common source in the auditory cortex; this interpretation is further supported by the different peak-latency changes as a function of the interstimulus interval (Fig. 5, right bottom panel). Thus, the auditory 100-ms response has likely (at least) two sources: a modality-specific source located in supratemporal auditory cortex, and a second source closer to the vertex, possibly located in the supplementary motor/sensory cortex; see Hari and Puce, 2017, pages 205–207, for a detailed discussion of the original data by Hari et al., 1982, Tuomisto et al., 1983.

In research settings, it may be laborious to apply EEG electrodes to all subjects completing an MEG recording, but in clinical examinations, there is no reason not to always record EEG with MEG, especially in epilepsy patients (Lopes da Silva, 2008, Stefan and Trinka, 2017). Naturally, the respective signals should be recorded using the same bandpass filters and sampling rates. Eventually, it is almost always useful to compare the MEG and EEG results.

In the combined use of MEG and EEG, the minimum requirement is that the MEG and EEG data should not contradict one another; if this were to be the case, one would have to carefully scrutinize the data further for artifacts and other possible issues. For further discussion of the relative pros and cons of MEG and EEG as far as equipment, sensitivity to currents of different orientations and sites, source estimation, etc., are concerned, see Hari and Puce, 2017, Baillet, 2017.

### 2.9 Group-level data

Clinical MEG recordings always aim to provide information that is valid for an individual patient. When various patient populations are studied and when laboratory-specific reference data are collected, it is, however, also necessary to summarize the results at group level.

Some sensor-space data can be clinically useful, especially because the analysis is fast, but source-space analysis typically provides many benefits. First, as sensor positions are not fixed, the head can move freely under the MEG sensor array, requiring either adjustment with motion compensation methods, or at the very least, confirmation of minimal head displacement (Gross et al., 2013a). Second, due to the field spread across the sensor array, functional connectivity analyses can yield erroneous results that manifest as inflated measures of correlation and coherence (Schoffelen and Gross, 2011, Zhang et al., 2014).

Analysis of MEG data in source space avoids some of the above-mentioned issues, and even grand-average source waveforms can be computed for a group of subjects. However, this process requires either volumetric (Evans et al., 2012) or (cortical) surface-based (Fischl et al., 1999) normalizations similar to those used in the analysis of fMRI data. Any kind of reports of group-level data in tabular or figural form, at the very least, should include the mean and a measure of variability (such as standard deviation, the standard error of mean, or the use of boxplots in figures). It is also recommended that the individual data points in figures be displayed, as this can provide additional information about the underlying distribution of the data across the groups being compared.

For statistical analysis of group-level MEG data, see the practical guidelines by the MEG community (Gross et al., 2013a).

## 3 Established clinical applications

### 3.1 Epilepsy

The first MEG identification of the generation site of epileptic spikes required a 16-h recording with a single-channel neuromagnetometer (Barth et al., 1982). Two decades later, the development of commercial whole-scalp MEG systems made it possible to conveniently and non-invasively record brain activity with high spatial density (300 sensors or more) and high temporal resolution (even 10,000 samples per second per channel), and to accurately locate the sources of those signals even in patients with gross anatomical distortions or skull defects caused by previous surgery or injury. Indeed, MEG has become part of the standard of care at epilepsy centers, utilized frequently to guide the implantation of intracranial electrodes (Sutherling et al., 2008, Knowlton et al., 2009, Jung et al., 2013, Murakami et al., 2016).

The main questions to be asked in the study of epileptic patients are whether there is a single epileptic focus or multiple foci, and what are their precise locations and temporal activation orders within the brain. In case of multiple foci, MEG’s high temporal resolution often allows the demonstration of consistent time lags that would imply an activation sequence, for example a primary focus in one hemisphere and a mirror focus in the other. Here the spike onsets have the best localization value.

The simplest and most widely applied source model employed in the analysis of clinical MEG data is the single equivalent current dipole, which assumes that at a given time instant the salient brain activity is focal and restricted to a single brain region, or to multiple distant brain areas that each are modeled with a current dipole. In the analysis of interictal epileptic spikes, the head can be modeled as a spherically symmetric conductor and, after inspecting the magnetic field pattern for dipolar structure (see Fig. 5, top panel, for an example), the best-fitting equivalent current dipole is found by a least-squares fit. Typically a spherical model fitted to the local head curvature works well for locating superficial sources but more realistic forward models can significantly improve the accuracy of dipole localization in frontal and deep brain areas (Tarkiainen et al., 2003). The locations of the sources of several identified spikes are visualized in individual MRIs or on 3D surface reconstructions derived from them.

In addition to the inspection of the spatial distribution of the MEG data, the validity of the dipole approximation can be assessed by considering whether (i) the dipole amplitudes (source strengths) are physiologically feasible, (ii) the locations of the fitted dipoles are at, or close to, the cortex, and (iii) the dipole locations form a cluster (Van 't Ent et al., 2003). Moreover, (iv) the goodness of fit of the source model and the confidence intervals of the source locations provide information about the fit between the measured MEG distribution and that predicted by the dipole.

Epileptic spikes last for 20–200 ms, popping out of the ongoing background activity, being often clearly discernable in visual inspection. Time–frequency analysis (Tallon-Baudry and Bertrand, 1999) shows the maximum power of spikes in the 20–70 Hz range, with power increases within −200 to +200 ms with respect to the spike, which has proven useful for volumetric imaging of the underlying sources (Bouet et al., 2012).

In the case of frequent spikes of similar morphology, one can average them because they likely arise from a single focus; here one can apply either template matching or trigger the averaging on the basis of thresholded amplitudes close to the peak values. Averaging multiple spikes improves source estimates as has been shown by comparing locations of MEG and intracranial spikes (Wennberg and Cheyne, 2014). When the spikes differ in morphology but still seem to be generated in the same region, one can examine the clusters of the sources of all spikes, provided that the SNR of the spikes is reasonable. A tight source cluster with some scatter often reliably refers to a single underlying epileptogenic area. However, as the spread of the cluster can be due to superimposed noise, it cannot indicate the extent of the source area (Bast et al., 2006).

Because of its sensitivity for identification of epileptic spikes (Lin et al., 2003, Iwasaki et al., 2005, Kakisaka et al., 2012), MEG has been used not only for localization of epileptic sources, but for diagnosis of epilepsy (Colon et al., 2009, Duez et al., 2016, Colon et al., 2017), especially when the results of other non-invasive studies have been meager or completely unrevealing. Although the dogma that MEG cannot see radial currents is widespread, less than 5% of the cortical surface is within 15 degrees of radial (Hillebrand and Barnes, 2002).

While localizable seizures occasionally occur during MEG recording, the observed epileptic MEG abnormalities are usually interictal. For ictal MEG, time-locked video–MEG recordings of the clinical manifestations of the seizure are essential for the association of the MEG signals with seizure semiology. How well the location of interictal spikes reflects the source of the patients’ seizures is a question that has vexed the EEG community for half a century. MEG and EEG are equally poor in differentiating between “red versus green” spikes, i.e., whether interictally observed spikes are or are not important for seizure generation. As in EEG, the occurrence of epileptic abnormalities can be increased by hyperventilation, photic stimulation, sleep, sleep deprivation, and some medications.

During the years, the recording and localization of interictal epileptic discharges and ictal events, especially for pre-surgical planning, has become the most important clinical application of MEG (Stefan et al., 2011, Kharkar and Knowlton, 2015). MEG can confirm a patient’s suitability for epilepsy surgery. The spatial resolution of MEG and the ability to separate nearby sources (Romani et al., 1982, Gavaret et al., 2014) are critical advantages of MEG in the refinement of the epileptogenic zone. In patients with focal epilepsy, the spiking volume determined by MEG overlaps in space with the seizure onset zone determined by intracranial recordings of spontaneous seizures. MEG has proven helpful for selecting good candidates for epilepsy surgery when structural brain MRI is negative (Jung et al., 2013) and for localizing the seizure onset zone, and thus planning the surgical resection in patients with focal cortical dysplasia (Bouet et al., 2017).

The yield of epileptic spikes has been much higher in MEG than in scalp EEG recordings (Iwasaki et al., 2005, Kakisaka et al., 2012), leading to a better sampling and localization of the epileptogenic zone. Accordingly, MEG has been found more effective than EEG in epilepsy screening (Ossenblok et al., 2007).

Identification of “MEG-unique” spikes (i.e., those with no correlate in the simultaneously recorded EEG) (Kakisaka et al., 2012) is especially valuable as it may uncover a previously unsuspected epileptic region of the brain or prompt re-examination of other imaging modalities to confirm another abnormal region. Furthermore, the sites and propagation of epileptic activity obtained with spatiotemporal source analysis agree better with intracranial EEG when the MEG rather than surface EEG is employed in the analysis (Tanaka et al., 2010).

To capture a rapid change or propagation of epileptic discharges after ictal onset, more advanced source modeling approaches are needed. One possibility is to create a multidipole model including several sources with fixed locations, but with different time courses. Such models can, for example, indicate timing differences between the initial focus and subsequent activity, which provides important information for surgical planning. In addition, distributed cortical source models have shown utility in following the evolution of the activity or telling when the activity is widespread rather than focal (Shiraishi et al., 2005). Both dipole models and cortically constrained distributed source estimates can be used in conjunction with both MEG and EEG and compared with other imaging information, as well as with the results of invasive recordings (Tanaka et al., 2010).

### 3.2 Pre-operative evaluation

In preoperative evaluation, the main tasks are (1) to identify the brain areas to be spared by the resection of a tumor or an epileptic focus relative to functionally identifiable landmarks, and (2) to map putative functions in the to-be resected region.

In the workup of patients under consideration for epilepsy surgery, MEG provides complementary information. The MEG findings can for example change the plan for intra-cranial electrode implantation and alter the surgical plan itself (Sutherling et al., 2008, The AAN Board of Directors, 2009, Knowlton et al., 2009).

Eloquent areas can be identified using different sensory stimuli (that activate, e.g., sensorimotor, auditory and visual cortices; see Mäkelä et al., 2001), motor tasks, or verbal/language stimuli. For that purpose, the source areas must be superimposed on individual MRI surface renderings (or brain sections). Plotting the vasculature on the same image serves as an important navigational aid for the neurosurgeon, and various 3D views to the surface and depth of the brain may be computed.

Examination of the individual brain anatomy, and the identification of major cortical landmarks, is very useful in this task as well. For example, Fig. 4 shows the anatomical identification of the central sulcus.

#### 3.2.1 Language function

Pre-operative mapping of language (and other brain functions) is routinely performed prior to resection of putative epileptogenic foci and/or neoplasms. Traditionally, electrocorticography (ECoG) is used together with direct electrical stimulation of the brain underlying the invasive electrodes to locate cortex devoted to sensorimotor function, language, and memory. These procedures are carried out acutely in the operating room, or chronically in a long-term epilepsy monitoring unit (or neurosurgical intensive-care unit).

Language-sensitive cortex is extensive and bilateral, although the main activation sites are located in the dominant hemisphere (Salmelin et al., 1994, Hickok, 2009). This bilaterality is at odds with the unilateral results of the Wada test (with intracarotid amobarbital procedure, IAP) that has traditionally been used to preoperatively evaluate language dominance (Wada and Rasmussen, 1960) and lateralization of verbal memory (Milner et al., 1962). The effects of amobarbital are short-lasting but long enough for quick testing of language dominance on the basis of stopping, slowing, or slurring of speech. Instead, only a cursory test of memory can be performed during the influence of amytal (Papanicolaou et al., 2014).

Until quite recently, the Wada test and ECoG with electrical stimulation were regarded as the “gold standards” for pre-operative assessment of epilepsy- and tumor-surgery patients. However, these gold standards have been repeatedly questioned in the literature (see Papanicolaou et al., 2014). Specifically, the Wada test’s role and the importance of language lateralization is decreasing in preoperative evaluation because fMRI can be used to easily map the entire circuit involved in language processing. Current opinion favors non-invasive methods, considering the Wada test and cortical stimulation to be no longer necessary for assessing epilepsy-surgery patients (Mathern et al., 2014).

In an MEG study used for determining language lateralization in patients prior to surgery on the basis of distributed source analysis (Tanaka et al., 2013), MEG agreed with the IAP results in 32 out of 35 patients. Several studies have also shown a good concordance of language laterality between MEG and the IAP by using a single dipole model (Papanicolaou et al., 2004, Merrifield et al., 2007, Rezaie et al., 2014).

TMS is increasingly used to complement or influence MEG-related examinations (Vitikainen et al., 2009, Mäkelä et al., 2015, Pathak et al., 2016, Albouy et al., 2017) and as a stand-alone application in language mapping (Krieg et al., 2017). TMS pulses can interrupt articulation, but the stimulation site does not necessarily identify the brain area supporting this function, as the pulses can block the transmission of signals along a neural pathway in the articulation circuitry.

When language function is assessed by identifying sources of auditory responses (Papanicolaou et al., 2004, Rezaie et al., 2014), or by recording event-related changes in oscillatory brain activity (Kim and Chung, 2008, Hirata et al., 2010), a “laterality index” (LI) may help to quantify the results. LI is a measure of hemispheric dominance, defined as the difference between the left- and right- hemisphere signals (MEG, EEG, or fMRI) divided by their sum: LI = (L – R)/(L + R). A more complex alternative to the LI has been proposed recently (D'Arcy et al., 2013).

At the time of writing, there is no agreed-upon standardized paradigm for testing language function or for evaluating verbal memory with MEG and we, thus, cannot yet give guidelines for such studies.

## 4 Clinical applications on the horizon

### 4.1 Stroke

A stroke in the territory of the middle cerebral artery typically causes deficits in motor and/or somatosensory circuits and impairs interactions between these two systems. Due to altered brain connectivity, some of the symptoms after a stroke can arise from brain areas remote from the damaged tissue. Deficits in sensorimotor integration impair both gross movements and fine-motor skills. MEG is well suited for investigating neurophysiological changes after stroke because it, unlike MRI, is independent of hemodynamic alterations; moreover, the passage of signals is practically unaffected by the morbid tissue.

In patients studied chronically post-stroke, MEG has demonstrated focal slowing in the perilesional tissue, as assessed with power-spectrum analysis (Butz et al., 2004) as well as reduced complexity of activity as assessed by a measure of entropy (Chu et al., 2015). The situation is similar to findings in brain-tumor patients in whom both theta and delta activity can have localizing value as they occur in cortex adjacent to tumors and in surrounding edematous cortical areas (Oshino et al., 2007).

Abnormalities of somatosensory evoked fields (SEFs) in response to either electrical or tactile stimulation can identify disease- and recovery-related changes in neuronal processing in either SI or SII cortices, or both, after stroke. For example, normalization of SEFs was associated with the recovery of hand functions (Rossini et al., 1998, Gallien et al., 2003, Tecchio et al., 2006, Roiha et al., 2011, Forss et al., 2012). As SEFs are highly reproducible and can be recorded without cooperation of the patient, they are well suited for studies of acute stroke patients (Forss et al., 1999, Wikström et al., 1999).

Spontaneous brain oscillations are also altered after stroke, as is well known from both EEG and MEG recordings (Tecchio et al., 2007, Galovic et al., 2018). Stroke induces bilateral changes in cortical excitability, likely associated with brain reorganization. These changes can be revealed by monitoring the modified reactivity of the ∼20-Hz oscillatory motor-cortex rhythm to tactile stimulation and passive movements, with altered excitability associated with recovery of hand function (Laaksonen et al., 2012, Parkkonen et al., 2015, Parkkonen et al., 2017).

MEG recordings in severely ill acute stroke patients are demanding as the co-operation may be poor and the patients are still in relatively unstable condition. A trained nurse, or a neurologist/physician should be present during the early post-stroke measurements. The patients can be studied while they are either in a sitting or a supine position but the sitting position is preferred, as it prevents the patients from falling asleep. Whenever possible, continuous head-position monitoring should be used.

### 4.2 Chronic pain

Neuropathic pain results from injury to nociceptive pathways and is associated with a reduction of pain evoked potentials (see Mauguière and Garcia-Larrea, 2018, for a review). In a recent review, Ploner and May (2017) concluded that MEG's advantage over EEG in pain research is its higher spatial resolution that makes it well suited for source localization; however, the authors emphasized the use EEG because of its affordability, accessibility, and mobility.

In chronic-pain patients, MEG findings of clinical value include maladaptive plasticity and its association with experienced pain intensity in phantom pain (Flor et al., 1995) and in complex regional pain syndrome (Juottonen et al., 2002; Maihöfner et al., 2003).

Pain-evoked magnetic fields should be suitable for assessing opercular–insular pain syndrome resulting from para-sylvian lesions (Garcia-Larrea et al., 2010) but, to our knowledge, no such study is hitherto available. Numerous MEG studies, based on source-space approaches, show changes in resting-state activity and functional connectivity in patients suffering from various types of chronic pain including migraine (Li et al., 2016, Xiang et al., 2016), menstrual pain (Kuo et al., 2017), and fibromyalgia (Lim et al., 2016, Hsiao et al., 2017). Deciphering whether these MEG markers might be useful to guide non-pharmacological treatments of chronic pain remains to be solved in the future.

### 4.3 Traumatic brain injury

So far, we are sorely lacking reliable and objective diagnostics of mild and moderate traumatic brain injuries. In traumatic brain injury (TBI), abnormally large amounts of 1–4-Hz activity have been recorded, resulting in 87% success rate for the detection of TBI patients (Huang et al., 2016). Thus, MEG could allow to identify mild and moderate TBIs even in the absence of macroscopically visible structural changes (Lee and Huang, 2014). Source estimation of MEG signals with respect to individual brain anatomy, obtained from MRI, could be the way forward for identifying injured patches of cortex, with an accuracy and precision that were not possible earlier.

### 4.4 Parkinson’s disease

MEG has been used as a research tool in Parkinson’s disease (PD) to study oscillatory network dynamics underlying or associated with rest tremor (Hirschmann et al., 2013a), akinesia (Hirschmann et al., 2013b), and cognitive performance (Olde Dubbelink et al., 2014). More recently, MEG recordings have also been combined with deep brain stimulation to reveal modulation of synchrony within distinct resting-state networks (Oswal et al., 2016). These studies often combine MEG with hand EMG and sometimes also recordings of local field potentials from deep brain structures, which complicates the studies methodologically. Although MEG is currently not yet applied as a clinical neurophysiological tool in PD, it may in the future become useful in the diagnosis and management of PD and other neurodegenerative diseases.

Few studies have applied evoked MEG responses to explore function of auditory and somatosensory cortices in PD and the effects of PD treatment on these functions (Pekkonen et al., 1998, Airaksinen et al., 2011, Sridharan et al., 2017). However, as with previous evoked potential studies, the results were at this stage either normal or not conclusive, or not explored enough to be useful for clinical applications.

### 4.5 Hepatic encephalopathy

Hepatic encephalopathy (HE) is a complex neuropsychiatric disorder resulting from acute or chronic liver disease. Depending on the disease stage, the clinical symptoms range from minor attentional deficits and motor impairment to severe cognitive disturbances and coma. MEG studies of HE—that so far have been limited to very few centers—have revealed HE-stage-dependent slowing of spontaneous and stimulus-induced oscillatory activity across different frequency bands and across different cortical systems (Butz et al., 2013). Frequency of cortex–muscle coherence is reduced in HE patients, which corresponds to the emergence of the typical tremor-like mini-asterixis. While these results have advanced the pathophysiological understanding of HE at a group level, more studies are needed to establish MEG as a useful neurophysiological tool to help diagnose and monitor individual patients with HE.

### 4.6 Neuropsychiatric disorders and dementia

MEG has been used to investigate alterations in brain dynamics associated with various neuropsychiatric disorders, including dementia. While many disorders are associated with alterations in evoked responses and brain oscillations, the selectivity in terms of disease is far from well-investigated. For instance, it has been demonstrated that the severity of depression can be predicted on the basis of diminished posterior alpha oscillations (Jiang et al., 2016); however, it remains unclear if this effect is selective to depression. Some progress has also been made in quantifying with MEG the connectivity in Alzheimer’s disease. For instance, alterations in the behavior and connectivity in resting-state networks as well as differences in auditory gating have been found to be associated with Alzheimer’s disease (Engels et al., 2017, Josef Golubic et al., 2017). The growing trend of collecting and analyzing ‘big data’ should aid in evaluating and developing the diagnostic potential of MEG for various disorders.

### 4.7 Brain maturation

MEG is, due to its non-invasiveness, a promising technique to study early brain maturation and to assess early signs of developmental disorders. The results could potentially lead to new intervention techniques for children at risk of developmental problems (Nevalainen et al., 2014).

Well-fed newborn babies and infants are often sleepy and therefore relatively easy to study with MEG. From the movement-artifact point of view, the most difficult age is from 6 months to 3–4 years; older children can often be motivated to stay still if they are allowed to view a video or are otherwise very well prepared for the examination. Some laboratories acclimate children to MEG by using a mock MEG helmet. Despite these precautions, movement artifacts and changes of head position complicate all developmental studies.

An extra challenge with the youngest children, and especially with premature babies, is their small head size, so that in adult MEG devices the distance from the neural currents to most of the sensors is several centimeters larger than in typical adult measurements. However, one hemisphere at a time can be easily positioned close to the sensor array because the entire head and shoulders of young infant will fit into the adult helmet (Shibata et al., 2017). Recently, MEG instruments have been developed with geometry optimized for infants (Roberts et al., 2014, Okada et al., 2016).

Some MEG responses can already be recorded from the fetal brain if loud sounds are delivered through the mother’s abdominal wall (Blum et al., 1985, Wakai et al., 1996, Draganova et al., 2005). At post-partum, as the child grows older, the latencies of the evoked response become shorter in all sensory modalities, and interestingly the polarities of some responses can change during infancy early childhood (Paetau et al., 1995, Lauronen et al., 2006), most likely mainly due to increasing myelination and possibly also because neurotransmitter systems may change during maturation.

Brain development occurs rapidly during the first years of life, and the process of adapting to the statistically dominant speech sounds in the environment results in discriminative responses already in neonates and infants (Imada et al., 2006, Bosseler et al., 2013, Kuhl et al., 2014); see also an early feasibility study of infant MEG recordings (Cheour et al., 2004). In 7-year-old children, the activation sequences start to resemble those in adults although still with longer response latencies (Parviainen et al., 2006).

Until now, mainly healthy children or patient groups have been studied, and thus the clinical utility of MEG recordings in the diagnostics and follow-up of individual pediatric patients remains to be shown.

## 5 Practicalities of clinical MEG recordings

### 5.1 General

Preparation of the patient for MEG recordings and taking the measurements includes several steps, and the following issues must be considered.

#### 5.1.1 Subject

(a) The patient and clothing must be non-magnetic. Demagnetizing (degaussing) using a hand-held alternating-current degausser can be helpful in decreasing any residual magnetic field.
(b) To avoid additional magnetic contamination, MEG should be completed before performing an MRI, whenever possible.
(c) It may be useful to measure head size (especially in children) before the recording, or to use a plastic replica helmet to test whether the head would fit into the MEG helmet. Remember that the EEG electrodes also take up space within the MEG helmet.
(d) The head should be centered inside the helmet, as close to the top and back walls as possible. This can be difficult/impossible for small heads, unless specialized pediatric MEG systems are used. Nevertheless, MEG recordings from infants are reliable and of localizing value when carried out with standard MEG equipment, without any special adaptation for small heads (Shibata et al., 2017).
(e) Acutely ill neurological (e.g., stroke) patients can suffer from neglect syndrome. Such patients are easier to measure in supine position, with the head supported tightly against the helmet (for example with tiny cushions). However, we recommend that patients with lowered vigilance are measured in sitting position to keep them more alert.
(f) The patient must be able to sit still and remain (relatively) immobile throughout the measurement. During the recording of early sensory (non-visual) responses, the patient can be reading or looking at a movie at the same time to maintain stable vigilance. Recording of long-latency responses typically requires more co-operation as the patient may need to be alert and/or to pay attention to the stimuli
(g) During major seizures, such as generalized tonic-clonic events, MEG recordings are contaminated by muscle and movement artifacts. Focal seizures, on the other hand, will often have many seconds of electromagnetic discharges before any clinical movements occur, permitting localization of the seizure onset zone. Interictal events can usually be captured without movement artifacts.
(h) Experiments in a metallic shielded room pose extra challenges regarding acoustic and electrical noise, and electrical safety; see, e.g., Hari and Puce (2017).

#### 5.1.2 Recording personnel

(i) Recording personnel should behave compassionately and efficiently, and inform the patient properly to minimize anxiety before the measurement. Consequently, good-quality data will be recorded.
(j) Recording personnel should have personal experience in being a subject for an MEG measurement to fully understand what it requires to stay immobile for long periods and to be isolated from the outside world in a magnetically shielded room. They should also be familiar with the institution’s and MEG unit’s health and safety procedures, in case of emergencies.
(k) Recording personnel should have basic knowledge about the generation and appearance of both brain signals and possible artifacts, so that artifacts can be minimized and carefully noted during the recording.
(l) Trained medical personnel should be present during measurements of acutely and/or seriously ill patients.

#### 5.1.3 Running the experiment

(m) Before commencing the recording, data should be available from empty-room measurements using a setup otherwise identical to that in the real MEG recording, but without the subject.
(n) Test measurements made before the real recordings can identify if some magnetic material is still in/on the patient, or whether the patient would need non-magnetic eye-glasses (if required to focus and fixate the eyes).
(o) Communication with the patient should be possible at all times via video monitoring and a two-way intercom.
(p) For the patient's comfort and alertness, the recording should be kept as short as possible, while allowing enough good-quality data to be collected. Epochs longer than say 10 min will easily lead to dampened evoked responses, increase of alpha-range spontaneous activity, and increased eye blinks and head movements, as well as poorer compliance to the task instructions. For the same reason, it is not recommended that a full session lasts longer than 1–2 h, and in most clinical studies the maximum duration is 1 h. The exception to this recommendation would be a sleep study.
(q) Good note-keeping is a must in all MEG recordings (like in any other clinical neurophysiology recording). Artifacts, the patient's level of cooperation and any changes in the patient's state should be noted, especially as another person may analyze the data, and even at a much later time.

#### 5.1.4 Data analysis

(r) Identify and omit from the analysis “bad channels” that contain large noise or clear artifacts.
(s) To improve the reliability of amplitude measurements and of field patterns based on the amplitude data, use stable baselines that are of sufficient duration.
(t) Instead of relying only on the coordinates of source locations, compare measured and predicted field patterns to find out whether the model should be modified.
(u) Note that the goodness of fit of a source model depends on many factors besides the appropriateness of the model: for example, the type and the number of channels included in the computations. Thus, the goodness-of-fit values are most useful for comparison of models with an equal number of parameters.
(v) An error in the depth of a source will always be accompanied by an error in the estimated source strength: the deeper the source, the stronger it appears to be. Thus, while evaluating source strengths, also pay attention to the source depths.

### 5.2 Clinical reports of MEG recordings

The conclusions of a clinical MEG examination can be based only on reliable responses, and thus the replicability of the measured signals should be first carefully checked and confirmed. Other physiological signals recorded concurrently with the MEG data can help separate out artifacts from real brain activity and potentially highlight some unique and novel findings.

The format of the clinical MEG report depends largely on the lab and local practice, but in general it is very similar to EEG and evoked-response reports. Typical information to be included (preferably in a template report) includes:

– Patient name, ID, gender, age, handedness, clinical background or diagnosis, and current medications potentially affecting the results.
– The recording and stimulation equipment (including software and version).
– Preprocessing pipeline and a comment on data quality.
– Stimulus specifications, such as intensities, physical qualities, ISI, visual angles of stimuli (e.g., check size and the entire stimulus).
– Filter settings.
– The number of averaged responses and a rough estimate of the number of responses that were rejected due to artifacts.
– Visual acuity and vision correction, as well as hearing threshold when relevant.
– Limb length and skin temperature when relevant.
– Description of background activity, its frequency composition, regularity, possible lateralization, and the occurrence of any neurophysiological abnormalities, such as epileptic discharges.
– The peak latencies and amplitudes and the sources of evoked responses compared with normative values (from the same laboratory).
– If appropriate, laterality indices (see section 3.2.1 Language function) across the hemispheres.

The discussion of the inference of the results, relative to the clinical diagnosis and the overall interpretation will vary by country and laboratory.

### 5.3 Experimental setups for different applications

#### 5.3.1 General rules and recommendations

Irrespective of whether spontaneous activity or evoked responses are recorded, a number of important and also simple additions to the routine recording protocol can help ensure that optimal quality MEG data are collected during the clinical recording session. Below we list some procedures that should precede any recording:

– Generate a set of normative values for every protocol that is used in the laboratory, so that data from individual patients can be referenced to these values.
– Record stimulus triggers in the same file as MEG data (for off-line analysis), and measure (and not only deduce) trigger–stimulus lags for all setups.

Always check the setups without connecting the patient to the stimulators (for example, the tactile stimulator should operate normally but not touch the patient), to be sure that the measured responses are not due to, e.g., auditory contamination.

– Measure head position *either* before (and after) each run *or* use continuous head-position measurement (especially in small children and in restless adults).
– Note that if the patients are keeping their hands on a table, movements made with one hand can be transferred as tactile stimuli to the other hand, resulting in artifactual responses in somatosensory cortex (Hari and Imada, 1999). This situation should, thus, be avoided if at all possible.
– Another (demagnetized) person in the shielded room with the patient should not touch the dewar or other parts of the MEG system, nor move around on the floor.
– Always measure both vertical and horizontal EOG (typically using EEG electrodes).
– Whenever feasible, collect spontaneous data where the patient is resting with eyes open for at least 2 min and eyes closed for 2 min in addition to recording evoked responses.
– Monitor both spontaneous activity and evoked responses online.
– Check, identify, and report artifacts online.
– Reject artifacts online on the basis of EOG-channel deflections (indicating eye blinks or large eye movements) and on the basis of large-amplitude changes on MEG channels.
– Save continuous raw data as they allow post-processing and additional analysis of the modulation of brain rhythms even in experiments where the main focus is on evoked responses. Typically, tSSS and other noise-suppression methods are used off-line.
– Check the replicability of evoked responses online during data acquisition by averaging the responses to two bins: responses to even-numbered stimuli to one bin and those to odd-numbered to another bin. Also collect replicates for the same condition in the beginning and end of the session whenever possible.
– Here, and in all evoked-response studies, avoid time-locking the stimulus interval to the phase of the power line; for example, in countries with 50-Hz power-line frequency (where one cycle is 20 ms), inter-stimulus intervals of 1005 ms are preferred to intervals of 1000 ms as they diminish the summation of the power-line artifact to the responses.

We will next briefly discuss special requirements of MEG recordings exploring the functions of sensory cortices.

### 5.4 Auditory system

#### 5.4.1 Background

Both middle-latency and long-latency auditory evoked fields (AEFs, MLAEFs and LLAEFs) can be easily detected from each hemisphere (for a review of the early steps of AEF recordings, see Hari, 1990). MLAEFs reflect activity of the primary auditory cortex in 50 ms or less following the stimulus. The early cortical deflections peak at around 19, 30 and 50 ms (named P19m, N30m and P50m to indicate that they are magnetic counterparts of the auditory evoked potentials, AEPs). P50m is sometimes also included in the LLAEF response, together with N100m, P200m and N250m. The 100-ms response (N100m or M100) likely arises from planum temporale just posterior to the primary auditory cortex. Unlike for EEG, recordings of brainstem auditory evoked responses with MEG are not clinically feasible because of the large number of trials that need to be averaged (Parkkonen et al., 2009).

Patient's hearing thresholds should always be checked prior to running the protocol, whether on the basis of an existing audiogram, or at a minimum by performing a hearing-threshold test with the stimuli to be applied during the MEG recording. In this way, the sound intensities can be customized so that they are delivered at the same level (in dB) above the hearing threshold across all subjects.

The ISI strongly affects the N100m response that saturates at around ISIs of 8 s (see Fig. 5, middle panel); in small children the recovery cycle is longer Depending on the stimulus repetition rate, one can record either transient responses (MLAEFs or LLAEFs) or steady-state responses (Romani et al., 1982, Hari et al., 1989, Gutschalk et al., 1999). Frequency tagging of the input of one ear at the time using steady-state responses (with repetition rates of about 20–40 Hz) can be used to document the transfer of signals from one ear to the auditory cortices of both hemispheres (Fujiki et al., 2002, Kaneko et al., 2003), which is not possible to by any other evoked-response recording.

#### 5.4.2 Indications

The main clinical applications for AEFs currently are the functional localization of the supratemporal auditory cortex in pre-surgical mapping and the examination of the effects of brain injury (e.g., stroke) on temporal-lobe function.

#### 5.4.3 Stimulation

– Monaural stimulation is preferred because during binaural stimulation significant central suppression takes place so that the cortical responses to binaural stimuli are far smaller than the sum of the responses to left- and right-ear stimuli (Tiihonen et al., 1989; Fujiki et al., 2002). To diminish variability related to successive recordings, the stimuli can be presented alternatingly to the two ears.
– For MLAEFs, optimal stimuli are clicks (about 1 ms duration) or brief tone or noise bursts. Their broad frequency content will generate a wide-spread stimulation of the basilar membrane in the cochlea.
– LLAEFs can be elicited by any abrupt sound onsets and even by changes within a long stimulus. For clinical purposes, optimal stimuli are brief 1-kHz tone bursts (e.g., 30 ms duration, 5 ms rise and fall times, about 60 dB above hearing threshold).
– Long-duration stimuli (lasting, e.g., 300 ms or longer) will also produce sustained fields.
– ISI can be about 1.5–2 s for LLAEFs and a few hundred milliseconds for MLAEFs.
– For steady-state AEFs, a wide range of stimulation frequencies can be used, typically around 20–40 Hz, but also considerably lower, e.g., above 5 Hz when the transient responses start to transform to steady-state responses. Clicks or very brief noise bursts are effective stimuli, and in frequency-tagging experiments, continuous sounds (tones, music, or speech) can be amplitude-modulated at different frequencies in both ears; the tag frequency can be found in the MEG signals both in time and frequency domains. It is important to avoid any interactions between stimulation frequencies (and their harmonic and subharmonic frequencies) between the two ears.
– White-noise masking of the opposite ear may be necessary if the hearing thresholds between ears are very different.

#### 5.4.4 Recording

– Passband 0.03–200 Hz, sampling rate at least 600 Hz.
– Average 40–100 responses for LLAEFs (with repetition) and 200–300 for MLAEFs.
– It is best that the patient keeps the eyes open to stay alert. A visual fixation point is useful so that eye movements are kept to a minimum.

#### 5.4.5 Data analysis

– An initial analysis period from –100 to 500 ms is typically sufficient unless sustained fields are recorded to long sounds. If needed, the final epoch length can be clipped post-hoc.
– The most common analysis consists of measuring the amplitudes and latencies of MLAEF P50m and LLAEF N100m and identifying their neural sources and hemispheric differences.
– Steady-state responses can be analyzed by averaging (e.g., in epochs of 2–4 cycles), by correlating with the periodic function at the stimulus repetition rate, or by using Fourier analysis. Amplitude or power (= amplitude squared) of the steady-state responses can be computed. Apparent, but not real, latencies can be determined for the steady-state responses (Hari et al., 1989).

#### 5.4.6 Interpretation and caveats

– Lastencies, amplitudes, source locations, source strengths, hemispheric differences, and interaural interactions are informative. For steady-state responses, only apparent, but not real, latencies can be determined.
– Earphones can transmit tiny signals to some MEG channels, and when correlating the auditory signal with brain activity, spurious correlations may arise. A recording in an empty magnetically shielded room using a polystyrene head wearing the earphones under the MEG helmet can identify channels most susceptible to this artifact.
– In source estimation, close-by sources activated at the same time can lead to confusing interactions.
– Auditory contamination may arise from other stimulation equipment and it is, thus, important that the recording and analysis personnel know well the expected waveform and spatial distribution of auditory responses (the same is true of course for measurements of all sensory modalities).

### 5.5 Visual system

#### 5.5.1 Background and indications

Visual evoked responses (VEFs/VEPs) can be used to assess lesions of visual pathways, and such recordings were popular (especially in multiple-sclerosis patients) before the availability of structural MRI. By selective stimulation of the visual field, the likely presence of prechiasmatic and retrochiasmatic lesions can be identified as prolonged latencies and reduced amplitudes of visual responses. Similar studies are still relevant for pinpointing white-matter pathology and post-stroke visual-field defects, such as hemianopia. Similarly, searching for compression in the visual pathways as a result of the mass effect of a nearby lesion, such as a tumor, can chart the status of the optic tract in question and assess recovery post-operatively. MEG’s advantage in the studies of the striate (primary visual) cortex is that it sees the mesial wall of the occipital cortex well (Nasiotis et al., 2017).

VEF deflections N75m, P100m and N145m to pattern reversal are generated in the lateroventral aspect of the calcarine sulcus, contralateral to the stimulated visual hemifield (Nakasato and Yoshimoto, 2000). In hemianopsia, P100m is abolished in the affected side (Nakasato et al., 1996).

The fusiform gyrus in the ventral stream is activated much more strongly by faces than by other stimulus categories (Halgren et al., 2000). Activity in the parieto-occipital sulcus is stronger for luminance stimuli relative to checkerboard patterns, and also does not appear to depend on the location of visual stimulation (hemifield or foveal/extrafoveal) (Portin and Hari, 1999, Portin et al., 1999). The visual-motion-sensitive cortex MT/V5 can be activated by various moving stimuli (Uusitalo et al., 1997).

#### 5.5.2 Stimulation

– Commonly used stimuli are pattern-reversal or -onset stimuli (e.g., checkerboards with ∼2 reversals or onsets per second) presented to the full visual field (>15 degrees, contrast of 75%), each hemifield and each of the four visual quadrants. Two check sizes (1 deg and 0.25 deg of visual angle) are commonly utilized.
– Flash or luminance stimulation (>20 degrees, rate <1.5 flashes per second) may be used if visual acuity has been severely compromised. For a standard on visual stimulus presentation in clinical VEP studies, see Odom et al. (2004).
– Faces, objects and words can be used as stimuli to study the ventral visual stream.
– Moving stimuli but also onsets of, e.g., checkerboard stimuli can elicit VEFs in MT/V5 area of the dorsal visual stream.
– Steady-state VEFs can be elicited from sinusoidal stimulation frequencies of 4 to 80 Hz, and the strongest responses peak at around 10, 20 and 40 Hz. Frequency-tagging experiments can also be performed, whereby different parts of the visual display are coded by different tagging frequencies of, e.g., dynamical noise (Parkkonen et al., 2008).
– A fixation cross is recommended for most clinical visual studies, unless the subject is allowed to freely gaze, for example at a movie (see, Lankinen et al., 2014).

#### 5.5.3 Recording

– Passband 0.1–200 Hz, sampling rate at least 600 Hz.
– Analysis epoch from −100 to 500 ms.
– Average around 100 responses (for each replication) to demonstrate the main deflections.

#### 5.5.4 Interpretation

– Peak latencies and amplitudes, lateralization, and source locations can be informative.
– If extrastriate responses (e.g., from the fusiform gyrus) are used clinically, it would be important to first document activity in calcarine cortex in response to checkerboard stimulation. In this way, any delays in the latencies of the VEFs could be properly interpreted because normal or delayed VEFs from the calcarine cortex would provide a context for interpreting the extrastriate VEFs.

#### 5.5.5 Caveats

– As VEF amplitudes can be severely reduced if stimulus edges are blurred, all VEF recordings should be performed while patients are wearing non-magnetic goggles corresponding to their regular corrective lenses. For stimulation of visual hemifields and quadrants, the patients need to fixate accurately on a central fixation cross.

### 5.6 Somatosensory system

#### 5.6.1 Background

Recordings of somatosensory evoked fields (SEFs) can demonstrate an orderly somatotopic organization in the primary somatosensory cortex (SI), especially for the generation sites of the early (19–60 ms) deflections elicited by electrical peripheral nerve stimulation. In the SI cortex located in the bottom and posterior wall of the central sulcus and in the postcentral gysus, SEFs to upper-limb stimulation mainly arise from tangential currents in area 3b whereas the neighboring areas 1, 3a, and 2 are less likely to contribute to the responses. However, for lower-limb stimulation many more cytoarchitectonic areas can contribute to the responses because currents in all SI subareas in the mesial wall of the hemisphere are tangential with respect to the skull; this anatomical organization is evident as a rotation of the field patterns as a function of time (Hari et al., 1996). Longer-latency SEFs arise from the posterior parietal cortex (PPC) and from the secondary somatosensory cortex (SII), but other sources exist as well (Mauguière et al., 1997).

PPC sources, which occur posterior and medial to hand SI, typically peak at around 70–110 ms, and the SII sources peak at 90–125 ms (with 10–20 ms longer latencies to ipsilateral than contralateral stimulation). The SII responses are much easier to detect with MEG than with EEG due to source orientation (Kaukoranta et al., 1986). For proprioceptive (passive movement) stimulation of the upper limb, the main deflections peak at 70–90 ms, with putative source locations in area 3b in the posterior wall of the central sulcus (Smeds et al., 2017).

Some somatosensory responses can be recorded even at about 600 Hz (Curio et al., 1994) and these high-frequency oscillations are abnormal in, e.g., patients with writer’s cramp (Cimatti et al., 2007).

#### 5.6.2 Indications

SEFs, combined with other measures, are useful for identifying the course of the central sulcus located just anterior to the sources for SI in area 3b. For this purpose, SEFs are typically measured by stimulating multiple body parts (e.g., face, hand, and leg.) Should a detailed map of the gyral and sulcal contributions of sources in SI be required, SEFs can be recorded together with their electrical counterparts—somatosensory evoked potentials—combined with high-resolution anatomical MRI.

In stroke patients, SEFs can provide information about disruptions of the cortical somatosensory network (SI, SII, PCC) involving both hemispheres (Forss et al., 1999, Forss et al., 2012). Studies of proprioceptive afference may also turn out to be clinically useful (Parkkonen et al., 2015) by allowing access to altered processing of proprioceptive information in various brain disorders, after limb inactivity after trauma, and in balance problems of peripheral origin in elderly people. However, robust clinical studies are not yet available.

Principles of SEF recordings have been reviewed previously (Hari and Forss, 1999, Kakigi et al., 2000, Hashimoto et al., 2004, Kakigi and Forss, 2010, Hari and Puce, 2017). For the corresponding evoked potentials, see for example Nuwer et al. (1994) and Mauguière and Garcia-Larrea (2018).

#### 5.6.3 Stimulation

– Electrical stimulation is delivered to distal peripheral nerves using monophasic electrical pulses of 0.1–0.3 ms in the upper and lower limbs (median, ulnar, radial nerves at the wrist or hand, and the posterior tibial and peroneal nerves at the ankle and lower foot). [Note that the nerves are stimulated (depolarized) at the site of the cathode (negative electrode)]. The intensity is adjusted to either exceed the motor threshold, or to be below the motor but above the sensory threshold. Fingers, toes, lips or tongue (facial nerve), or other body parts, such as the genitalia (pudendal nerve), can also be stimulated should clinical needs dictate so. For stimulation of skin and sensory nerves, the intensity is usually 2.5–3 times the sensation threshold. Electrical stimulation is typically used only for transient SEFs because high stimulus repetition rates can feel unpleasant and also cause painful tetanic contraction of the limb muscles during recording of steady-state responses. The best stimulation sites are discussed in texts of clinical neurophysiology (Cruccu et al., 2008, Mauguière and Garcia-Larrea, 2018).
– Use constant-current (rather than constant-voltage) pulses, typically 0.2–0.3 ms in duration. Avoid pulses as long as 1 ms as they directly stimulate the underlying muscles.
– Artifacts caused by electrical stimulation can be largely diminished by tightly twisting the wires of the stimulation electrodes and by avoiding large current loops (that would produce strong magnetic fields). Moreover, the wires should be kept as far from the patient as possible. Artifacts are most problematic for stimulation of the face (different branches of the trigeminal nerve), where it is impossible to satisfy this requirement. In this case using a wide-passband filter in the MEG recording will help ensure that the stimulus artifact does not bleed into the desired response latency range.
– The ISI can be 0.2–1.0 s for early SI responses, but should be increased to about 3 s for PPC and SII responses because of their longer recovery cycles (Hari et al., 1993b). In the latter case, alternating stimulation of the left and right body sides at 1.5 s (or to avoid 50-Hz contamination, 1.505 s) intervals would be the most time-efficient.
– Because PPC and SII responses are sensitive to changes in vigilance and attention, the measurements should be kept as short as possible, and the patient should be instructed to ignore the stimuli.
– Tactile stimulation activates rapidly adapting cutaneous mechanoreceptors and is, therefore, more natural and selective than electrical stimulation, which activates a variety of fibers (Johansson and Vallbo, 1979, Hashimoto, 1987, Forss et al., 1994). Tactile stimuli can be applied, e.g., by delivering air puffs to the skin surface or using a MEG-compatible pneumatic stimulator containing pressure-filled diaphragms attached to the finger tips (Mertens and Lütkenhöner, 2000). However, the slow stimulus rise in the latter stimuli prevents the earliest responses to be clearly delineated. Fortunately, these forms of stimulation are well tolerated even in children.
– A hand-held or machine-operated brush stimulator can be used to activate skin in any part of the body (Jousmäki et al., 2007).
– Proprioceptive afference can be elicited by passive-movements performed either by the experimenter (Bourguignon et al., 2011) or by using a computer-controlled pneumatic artificial-muscle device (Piitulainen et al., 2015). Accelerometers should be fixed on the limb that is passively moved, so that the movement excursion and timing can be accurately documented in the MEG data file that contains triggers and regressors for later analysis.

#### 5.6.4 Recording

– Passband 0.03–200 Hz, sampling rate of at least 600 Hz.
– Average about 100 responses for SI, and at least 40 for SII (with replications for both).
– For studies of proprioception, either transient or steady-state responses (corticokinematic coherence, CKC) can be collected (with sampling frequency, filters and number of signal averaging similar to those for the SEPs).

#### 5.6.5 Analysis

– When stimulus artifacts cannot be avoided (e.g., during trigeminal nerve stimulation), the signal at the time of the artifact can be zeroed out post-hoc but, as already mentioned, the recording passband has to be wide enough to prevent spreading of the artifact to latencies of interest. At this point, more standard, narrower digital filtering can be employed.
– Analysis epochs from –20 to 100 ms for SI response and from –100 to 400 ms for PPC and SII responses as well as for proprioception studies.
– The analysis of steady-state SEFs is similar to that described in the AEF section.

#### 5.6.6 Interpretation

– For median-nerve stimulation, the 20-ms response N20m around 20 ms indexes activity in the SI cortex. For foot stimulation, the earliest SI responses peak at around 40 ms.
– PPC responses peak at about 90 ms and SII responses peak at around 100 ms in both hemispheres, typically 10–20 ms later in the ipsilateral than contralateral hemisphere.
– The main responses to proprioceptive stimulation peak at 70–90 ms. Note that repetitive movements contain two phases (extension and flexion) with slightly different time courses and different proprioceptive afference, so that the frequency of the steady-state response, here also called corticokinematic coherence, is double compared with the movement frequency (as computed as full movement cycles).

#### 5.6.7 Caveats

– SI responses are quite resilient to changes in subject’s state and stimulus repetition but SII and other longer-latency responses can be considerably affected by subject's vigilance.

### 5.7 Pain

#### 5.7.1 Background

MEG is well suited to recording responses to painful stimuli in SII, and sometimes also in SI, whereas activations of anterior cingulate cortex and anterior insula are more difficult to detect with MEG. Despite considerable research in this area, pain-related MEG responses are not yet used systematically in clinical diagnostics or follow-up of individual patients although there is future clinical potential for the selective stimulation of A-delta and C-fibers.

The majority of functional brain imaging studies on pain have described cortical responses associated with A-delta-fiber-mediated pain, or a combination of A-delta and C-fiber pain. Selective C-fiber stimulation, although quite difficult, can be provided by using conduction blockade of A-delta fibers or by applying weak (2–4 J/s) temperature-controlled laser heat stimuli to a tiny (0.4 mm diameter) skin area (Bragard et al., 1996, Kakigi et al., 2003, Forss et al., 2005). The physiological basis for this stimulus selectivity is the higher density and lower activation threshold of the C- than A-delta fibers of the skin. Therefore, laser stimulation delivered to a tiny skin area with low total energy is likely to activate predominantly the unmyelinated C-fibers, often felt similar to so-called second or burning pain; however, some subjects report feeling only pressure, touch, or slight pain.

Noxious stimuli also affect the rhythmic activity that can be analyzed in either the time or frequency domain (Raij et al., 2004, Stancak et al., 2005).

More details are available in previous review articles on MEG recordings used in pain research (Kakigi et al., 2000, Hari et al., 2003, Kakigi et al., 2003, Kakigi et al., 2005).

#### 5.7.2 Indications

Although laser-evoked potentials are now accepted as the main technique to investigate and classify neuropathic pain syndromes (Cruccu et al., 2010, Truini et al., 2013), no clinical application of pain-evoked MEG responses has yet been validated for the diagnosis of chronic pain syndromes, in part because of limited access to MEG devices in clinical settings.

#### 5.7.3 Stimulation

– The ideal painful stimulus should be pain-fiber specific, controllable, safe, and reproducible. At present, three methods satisfy these criteria: painful laser stimulation (Forss et al., 2005), intracutaneous epidermal electrical stimulation (IES, Inui and Kakigi, 2012, Kodaira et al., 2014), and contact heat (Chen et al., 2001, Granovsky et al., 2016). The majority of functional brain imaging studies on pain have described cortical activation to A-delta-fiber-mediated pain using skin laser stimulation (Cruccu et al., 2008).
– Short painful laser pulses elicit prominent MEG responses. An assistant can direct the laser beam on a skin area of approximately 5 cm in diameter. To avoid skin burns and adaptation, the stimulus site should be moved after each pulse to a random direction in the selected skin area (typically in the dorsum of the hand). Stimulus intensity can be adjusted individually to equal twofold the subjective pain threshold.
– IES and laser stimulation can activate selectively A-delta and C-fibers. Both stimulators are commercially available and safe and easy to use, provided that manufacturer’s safety guidelines are adhered to.
– Contact heat used in EEG-based pain research and clinical studies produces strong artifacts in MEG environment, requiring specialized artifact rejection methods to be applied (Gopalakrishnan et al., 2013).

#### 5.7.4 Recording

– Passband 0.1–100 Hz, sampling rate 600 Hz.
– Average about 40–50 responses for A-delta and about 10–20 for C-fiber stimulation, depending on the SNR.
– Response amplitudes increase along with increasing ISI and the best signal-to-noise ratio during a fixed measurement time is achieved by using the optimum ISI (Raij et al., 2003); note, however, that the recovery cycles are different for responses to A-delta and C-fiber stimuli. For A-delta stimuli, SII response amplitudes increase strongly with ISIs from 0.5 to 4 s and saturate at ISIs of 8 to 16 s (Kakigi et al., 2005, Kakigi and Forss, 2010). The “ultra-late” C-fiber responses have even longer recovery cycles, and to avoid attenuation of responses due to habituation, the sessions should be kept short (Kakigi et al., 2003, Kakigi et al., 2005, Kakigi and Forss, 2010) but can be repeated after a break.
– Not only attention and vigilance, but also anticipation of pain may affect response amplitudes. The use of random ISIs (for example between 4–6 s) can decrease the anticipation effects.
– Always use EOG to monitor eye movements and blinks as they easily become time-locked to painful stimuli.

#### 5.7.5 Analysis

For A-delta responses the analysis period can be from −100 to about 400 ms whereas for C-fiber responses, the analysis epochs should be of at least 2 s for both upper- and lower-limb stimulation.

#### 5.7.6 Interpretation

The early deflections peak about 200 ms after laser stimulation and 160 ms after IES. The spatial patterns of MEG and EEG differ considerably for reasons that are not yet fully understood. MEG responses peak 10–20 ms earlier in the contralateral than ipsilateral hemisphere, with main generators in SII and insula.

Intra-cortical SEEG recordings have recently shown a matrix of 14 regions to respond to painful laser stimulation (Bastuji et al., 2016), and it is, thus, obvious that neither MEG nor scalp EEG can differentiate and identify all pain-related brain areas.

#### 5.7.7 Caveats

At present, we are still missing an “objective” indicator of the perceived pain.

#### 5.7.8 Safety issues

To avoid skin burns, the stimulus site must be slightly moved after each stimulus to a new place within a limited skin area, for example 10 cm^2^. A grid drawn on the stimulus site can serve as a visual aid for delivering the stimuli to different locations.

Both the patient and the assistant who handles the stimulator need to be protected with eye goggles to avoid possible injury if the laser beam is accidentally deflected into the eyes.

### 5.8 Motor system

While there is a reasonably large research literature on the slow event-related fields, such as the readiness fields and potentials (Bereitschaftspotentials) that precede voluntary movements, these signals have not become popular in the clinical sphere. Some reasons for this might be that they are rather difficult to record because of their slow time course and because they require good co-operation by the patient who has to make brisk and well-replicable movements. As an alternative one may monitor spontaneous sensorimotor ∼20-Hz oscillatory MEG rhythms that inform about the functional state of the motor cortex (Hari et al., 1998, Silén et al., 2000; Juottonen et al., 2002; Visani et al., 2006, Laaksonen et al., 2013). The 20-Hz oscillations initially decrease (suppression; event-related desynchronization, ERD) and subsequently increase (rebound; event-related synchronization, ERS) to tactile stimulation or movement (Pfurtscheller, 1981, Salmelin and Hari, 1994a, Hari et al., 1997, Salenius et al., 1997b, Neuper and Pfurtscheller, 2001).

The enhancements (rebounds) of the 20-Hz Rolandic rhythm indicate decreased excitability of the motor cortex, as assessed with transcranial magnetic stimulation (Chen et al., 1999, Takemi et al., 2013). Thus, alterations in dynamics of ∼20-Hz motor cortex oscillations may be useful to study the functional state of the motor cortex, e.g., post-stroke. Here just the envelope of, say, 15–25 Hz activity can be monitored.

The 20-Hz rhythm is bilaterally modulated to unilateral stimulation, but the modulation is stronger in the hemisphere contralateral to the stimulated hand (Salmelin and Hari, 1994a, Salenius et al., 1997b, Laaksonen et al., 2013). The 20-Hz oscillations are modulated also by passive movements, indicating that they are sensitive to proprioceptive afference (Piitulainen et al., 2013).

*Cortex–muscle coherence* (CMC) was discussed earlier and has been shown to be abnormal in several brain disorders, such as Parkinson’s disease and progressive myoclonus epilepsy. Both CMC and corticokinematic coherence (CKC) can be useful in future studies of motor function. CKC is especially attractive as it is very robust against magnetic artifacts (Bourguignon et al., 2016).

Studies of motor function, especially in patients, should include measures of the maximum force applied in the task (e.g., isometric contraction).

## 6 Future considerations

The dynamic field patterns and time courses of MEG signals provide rich temporal and spatial information. With the advent of large data bases (Niso et al., 2016) and the ever-improving machine-learning algorithms we can expect useful MEG-based biomarkers to emerge for various diseases. We can also look forward to reliable automatic analyses for clinical purposes to shorten the analysis times of, e.g., preoperative evaluation of epileptic patients.

Many experimental setups that are currently used for basic research of human sensory, cognitive, and social functions could already now be applied in clinical settings as well, and clinical applications of MEG should be taken into more wide use (Bagic et al., 2017, De Tiege et al., 2017). Ultimately more clinical applications will create more pressure for further development of MEG technology, which in turn will also benefit the broader neuroscience community. The new sensor technologies that are currently being tested will offer the prospect of more affordable, less maintenance intensive, more sensitive sensor arrays that may also become more easily movable.

One important future task for the MEG community is to develop evidence-based guidelines for clinical MEG applications that could be evaluated by Cochrane-type meta-analyses.

## Conflicts of interest

G. Barnes holds a Wellcome collaborative award that includes an intellectual property agreement with QuSpin Inc., a manufacturer of optically-pumped magnetometers. N. Nakasato is Professor and Chair of Donated Fund Laboratory from RICOH Japan Corp. and has received research funds and speaker's fees from Otsuka Pharmaceutical, Daiichi-Sankyo, UCB Japan, Fukuda Denshi, Pfizer Japan, Kyowa-Hakko-Kirin, and Eisai.

## Funding

R. Hari was supported by the Finnish Cultural Foundation (Eminentia Grant). S. Baillet was supported by a Discovery Grant from the National Science and Engineering Research Council of Canada (436355-13), the National Institute of Biomedical Imaging and Bioengineering (2R01EB009048-05), and a Platform Support Grant from the Brain Canada Foundation (PSG15-3755). G. Barnes acknowledges that The Wellcome Centre for Human Neuroimaging is supported by core funding from the Wellcome [203147/Z/16/Z]. N. Forss was supported by Helsinki University Hospital Research Fund and by the Finnish Funding Agency for Technology and Innovation (Grants No. 1104/10 and 1988/31/2015). J. Gross is supported by the Wellcome Trust (098433). O. Jensen is funded by the Wellcome Trust (207550). M. Hämäläinen was supported by the National Institute of Biomedical Imaging and Bioengineering (grants 5R01EB009048, P41EB015896, and U01EB023820). N. Nakasato was supported by JSPS KAKENHI Grant No. JP16H05435. A. Schnitzler was supported by the German Research Foundation (CRC 974). S. Taulu was supported by the I-LABS Ready Mind Project and a grant from the Washington State Life Sciences Discovery Fund (LSDF).
